# Bio-orthogonal chemistry-based strategy to Turn-OFF CRISPR-Cas9 activity in solution and live cells

**DOI:** 10.1093/narmme/ugag008

**Published:** 2026-01-30

**Authors:** Bhoomika Pandit, Sweta Vangaveti, Justa F Sentre, Ian McClain, Gabriele Fuchs, Maksim Royzen

**Affiliations:** Department of Chemistry, University at Albany, 1400 Washington Ave. Albany, NY 12208, United States; The RNA Institute, University at Albany, 1400 Washington Ave. Albany, NY 12208, United States; Department of Chemistry, University at Albany, 1400 Washington Ave. Albany, NY 12208, United States; Department of Chemistry, University at Albany, 1400 Washington Ave. Albany, NY 12208, United States; Department of Biology, University at Albany, 1400 Washington Ave. Albany, NY 12208, United States; Department of Chemistry, University at Albany, 1400 Washington Ave. Albany, NY 12208, United States

## Abstract

The CRISPR–Cas9 system has become a widely used gene-editing tool. Here, we present a new method for small-molecule control of CRISPR-Cas9 using bio-orthogonal chemistry between tetrazine (Tz) and *trans*-cyclooctene (TCO). We carried out molecular modeling studies and identified a unique position on single guide RNA (sgRNA) that can be site-specifically tagged with Tz without disrupting its activity. We also synthesized a series of TCO-modified CRISPR suppressors. When exogenously added, they bind to the Tz-tagged sgRNA, perturb the system, and drastically reduce the nuclease activity. The most successful suppressor is a TCO-modified six-amino acid-long cell-penetrating peptide, which shows excellent cell permeability. We showed that our method to control CRISPR-Cas9 nuclease activity is general by applying it to three different sgRNAs. We also showed that our method works in solution, as well as live HEK293 cells. We utilized flow cytometry to demonstrate the inactivation of the CRISPR-Cas9 system targeting GFP. Lastly, we showed the therapeutic potential of our method by targeting vascular endothelial growth factor A (VEGFA). Overall, the described method enables robust and minimally perturbing control of CRISPR–Cas9 nuclease activity using TCO suppressors to regulate Tz-modified sgRNA, targeting various genes *in vitro* and in live cells, offering a broadly applicable platform for controlled gene editing.

## Graphical Abstract

**Graphical Abstract:**
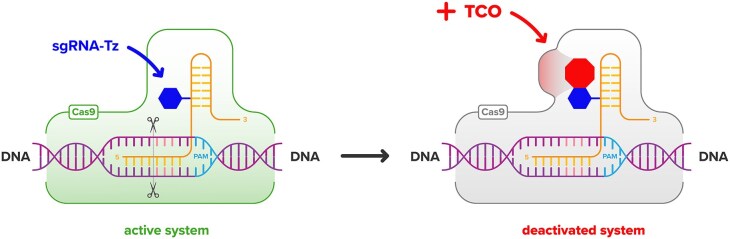

## Introduction

The CRISPR (clustered regularly interspaced short palindromic repeats)–Cas9 system has become a widely popular tool for genome engineering in different organisms and biological systems. The most frequently used system is the type II Cas9 from *Streptococcus pyogenes* strain SF370 (SpyCas9) and single guide RNA (sgRNA), which targets specific DNA sequences in the genome to create a blunt-ended double-strand breaks [1, 2]. In recent years, CRISPR technology has been evaluated in several human clinical trials to treat various cancers, eye disease, and chronic infection [3–5]. Perhaps the most striking biomedical breakthrough has been in the treatment of sickle cell disease, the most common inherited hemoglobinopathy worldwide [6, 7]. In 2023, the FDA approved a milestone sickle cell disease treatment, Casgevy, that is based on CRISPR technology.

Many ongoing clinical trials utilize lipid nanoparticles (LNPs) to co-deliver the two gene-editing components [8, 9]. The encapsulated sgRNA is typically heavily modified to enhance its stability and prolong its *in vivo* half-life. Meanwhile, Cas9 is co-delivered in the form of messenger RNA that encodes the endonuclease. One of the drawbacks of this strategy is prolonged expression of Cas9, which in combination with the stabilized sgRNA, can increase the so-called off-target effects [10]. Off-target editing is known to occur on a slower timescale than editing of the on-target sites [7, 11, 12]. Off-target editing can occur at unintended cleavage sites that have as many as five mismatches within the protospacer [13]. Therefore, there is considerable interest in developing systems that, in a controlled way, can turn off CRISPR-Cas9 nuclease activity. Such systems will inactivate either Cas9 or sgRNA once the target gene has been edited to prevent further editing of off-target sites.

A number of naturally occurring proteins capable of inhibiting Cas9 have been discovered [14–16]. The so-called anti-CRISPR proteins are utilized by phages to evade the CRISPR-Cas9 bacterial defense system. Meanwhile, controllable Cas9 nucleases have been achieved by various protein bioengineering strategies. For example, the Liu group inserted an evolved intein at specific positions in Cas9, making the resulting engineered nucleases responsive to a small molecule, 4-hydroxytamoxifen [17]. An alternative design was reported by Tan and coworkers who fused the Cas9 enzyme with the hormone-binding domain of the estrogen receptor (ERT2), also making it responsive to 4-hydroxytamoxifen [18]. Choudhary group fused Cas9 to the destabilized polypeptide domains of *E. coli’s* dihydrofolate reductase [19]. The stability and thus nuclease activity were controlled using a trimethoprim compound. Alternative methods to modulate Cas9 activity have been reported by the Yee group [20].

Analogously, a number of sgRNA constructs have been engineered to incorporate trigger-responsive domains. For example, Batey and co-workers incorporated theophylline-responsive aptamers into sgRNA, enabling small-molecule-controlled editing in E. coli [21]. Kundert *et al.* reported a different sgRNA design, containing theophylline and 3-methylxanthine-responsive aptamers. The reported constructs could be either activated or deactivated in a dose-dependent fashion in response to two different small molecules [22]. Tian group reported a small-molecule-responsive guide RNA, which was modified with ligand binding sites [23]. The authors used naphthyridine carbamate dimers to control the nuclease activity. There are also several reports describing light-responsive guide RNAs [24–29].

**Figure 1.**
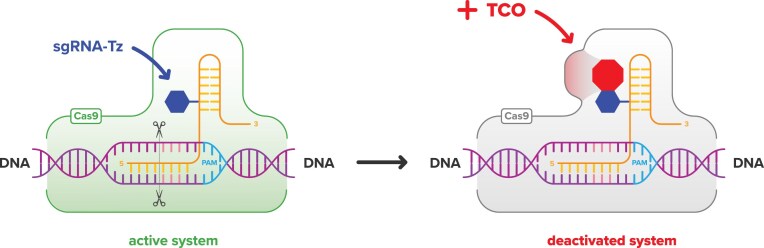
Schematic representation of the inactivation of CRISPR-Cas9 activity using bio-orthogonal chemistry between TCO and Tz.

Herein, we describe an approach to control CRISPR-Cas9 activity using small molecule bio-orthogonal chemistry between *trans*-cyclooctene (TCO) and tetrazine (Tz) [30]. The reaction between TCO and Tz has exceptionally fast kinetics. The two reacting groups are biocompatible and have high potential for bio-medical translation [31, 32]. The design is schematically illustrated in Fig. 1. In our design, sgRNA is modified with a single covalently attached Tz tag. Site-specific Tz tagging has been experimentally optimized to cause minimal perturbation to sgRNA’s function in Cas9-assisted nuclease activity. Thus, the construct shown on the left in Fig. 1 is constitutively functional. To turn off CRISPR activity, a TCO-containing small-molecule suppressor will be exogenously added. The TCO group of the suppressor will react via bio-orthogonal click reaction with the Tz group attached to sgRNA. The conjugation product, shown on the right in Fig.1, will cause perturbation in the domain of sgRNA–Cas9 complex that is important for nuclease activity, thus rendering the ribonucleoprotein complex inactive. In comparison to already reported methods to control CRISPR-Cas9 activity, the key advantage of our design is that it involves a small-molecule RNA tag, which minimally perturbs sgRNA’s structure, and small-molecule suppressors, which can be easily administered to control the nuclease activity. As we will discuss below, our design is general and does not require extensive protein or RNA bioengineering. The tag can be installed at a precise position of the guide RNA during the solid-phase synthesis. The design of small-molecule suppressors is modular and can be easily adapted to a particular application of interest.

## Materials and methods

All oligonucleotide solid-phase syntheses were done on a 1.0 μmol scale using the Oligo-800 synthesizer (Azco Biotech, Oceanside, CA, USA). Solid phase syntheses were performed on control-pore glass (CPG-1000) purchased from Glen Research (Sterling, VA, USA). Other oligonucleotide solid-phase synthesis reagents were obtained from ChemGenes Corporation (Wilmington, MA, USA). Phosphoramidites (TBDMS as the 2′-OH protecting group): rA was N-Bz protected, rC was N-Ac protected, and rG was N-iBu protected. A, C, G, and U phosphoramidites were dissolved in anhydrous acetonitrile (0.07 M) directly before use. The coupling step was done using a 5-ethylthio-1H-tetrazole solution (0.25 M) in acetonitrile for 12 min. The 5′-detritylation step was done using 3% trichloroacetic acid in CH_2_Cl_2_. The oxidation step was done using I_2_ (0.02 M) in THF/pyridine/H_2_O solution.

Post-synthesis, the oligonucleotides were cleaved from CPG and deprotected using 800 μL of AMA reagent (Ammonium Hydroxide/40% Methyl Amine in water) for 45 min at 65 °C. The supernatant containing cleaved and deprotected oligonucleotides was concentrated and resuspended in 100 μL of DMSO. Desilylation of 2′-OH groups was carried out upon addition of 125 μL of triethylamine trihydrofluoride (MilliporeSigma, cat# 344 648) and incubation for 2.5 h at 65°C. Lastly, crude RNA oligonucleotides were precipitated by adding 25 μL of 3.0 M NaOAc buffer (pH 5.5), followed by 1 mL of ice-cold absolute EtOH and incubation at -78 °C for 16 h.

For gel electrophoresis, 10X Tris/Borate/EDTA (TBE) buffer was purchased from Fisher Scientific Company L.L.C. (Waltham, MA, USA) and used with proper dilution. 30% Arcylamide/Bis-arcylamide solution (29:1) was purchased from Bio-Rad Laboratories, Inc. (Hercules, CA, USA). GeneRuler 1 kb Plus DNA Ladder (cat.# FERSM1331) was purchased from Fisher Scientific. Chromatographic purifications of synthetic materials were conducted using SiliaSphere^TM^ spherical silica gel with an average particle and pore size of 5 μm and 60 Å, respectively (Silicycle Inc, QC, Canada). Thin-layer chromatography (TLC) was performed on SiliaPlate^TM^ silica gel TLC plates with 250 μm thickness (Silicycle Inc, QC, Canada). Flash chromatography was performed using a Biotage Isolera One instrument (Biotage Sweden AB, Uppsala, Sweden). Preparative TLC was performed using SiliaPlate^TM^ silica gel TLC plates with 1000 μm thickness. ^1^H, ^13^C, and ^31^P NMR spectroscopy was performed on a Bruker NMR at 500 MHz (^1^H) and 126 MHz (^13^C). All ^13^C NMR spectra were proton decoupled. High-resolution ESI-MS spectra of small molecules were acquired using an Agilent Technologies 6530 Q-TOF instrument.

The peptides, **RRWQW** and **RLRWR**, were purchased from GenScript. They are based on the smallest-known cell-penetrating peptides (CPPs). PNAs were synthesized using Fmoc-Rink Amide AM Resin (Aapptec, cat. # RRZ001). PNA monomers, Fmoc-PNA-T-OH and Fmoc-PNA-A(Bhoc)-OH, were purchased from Biosearch Technologies (cat. # LK5004-B500 and LK5001-B500). PNAs were synthesized using the procedure described by Braasch *et al.* [33] Afterwards, **RRWQW, RLRWR**, and the PNAs were coupled to (*E*)-2-(Cyclooct-4-en-1-yloxy)acetic acid using procedures described in the Supporting Information (Supplementary Figs S3–S6). The resulting TCO-modified CRISPR suppressors, **TCO-CPP-RRWQW, TCO-CPP-RLRWR, AAA-PNA-TCO**, and **TTT-PNA-TCO** were purified by preparative HPLC using Phalanx C18 250x20 mm column (Higgins Analytical, cat. # PS-2520-C185).

### CRISPR-Cas9 *in vitro* DNA cleavage assay

pBR322 plasmid is a 4 361 base pairs long dsDNA (New England Biolabs, 1 mg/mL, cat.# N3033S). 1 μL of pBR322 plasmid DNA was diluted with water (16.87 μL) and NEBuffer^TM^ 3.1 (10x, 2 μL). The plasmid was linearized directly prior to CRISPR experiments using PvuII (10 U/μL, 1 μL, NEB, R0151S) for 1 h at 37°C. eGFP-N1 plasmid is a 4733 base pairs long dsDNA (Clontech, 1 mg/mL, cat.# 6085–1). 1 μL was diluted with water (16.87 μL) and NEBuffer^TM^ 3.1 (10x, 2 μL). The plasmid was linearized directly prior to CRISPR with DraIII-HF (20 U/μL, 1 μL, New England Biolabs, cat.# R3510L). For the Cas9-mediated DNA cleavage assay, sgRNA (300 nM, 5 μL), Cas9 (1 μM, 0.3 μL, New England Biolabs, cat.# M0386S), Cas9 buffer (10x, 1 μL, New England Biolabs), linearized plasmid (20 nM, 1.5 μL), and MQ H_2_O (2.2 μL) were mixed (final volume = 10 μL) and incubated for 16 h at 37°C. CRISPR experiments were terminated by the addition of proteinase K (20 mg/mL, 0.5 μL) for 1 h at 37°C. The reaction (10 μL) was mixed with blue loading buffer (6x, 2 μL, New England Biolabs, cat.# B7703S) and loaded on a 1% agarose stained with ethidium bromide (1x TBE running buffer). The agarose gel images were processed using BioRad Image Lab software (version 6.0), which is part of the BioRad Chemidoc MP Imaging System. DNA cutting efficiency was determined by quantitating the intensities of the top band (uncut DNA) relative to the intensity of the linearized plasmid. The reported DNA cutting efficiency values are averages of duplicate experiments.

### CRISPR-Cas9 experiments in HEK293 cells

CRISPR-Cas9 experiments were carried out following the procedure reported by Yin *et al.* [34] The GFP-expressing HEK293 cells were purchased from GenTarget (cat.# SC001) and cultured in DMEM, containing 10% FBS and 1X Penicillin/Streptomycin, at 37°C, 5% CO_2_, and 95% humidity. The cells were seeded at a concentration of 1 × 10^5^ cells per well in a six-well plate 24 h prior to the experiment. The cells were transfected with Cas9 mRNA (500 ng, Thermo Fisher Scientific), GFP-targeting **sgRNAs** (30 nM) using lipofectamine (1.5 μL) (Invitrogen™ LMRNA003) for 72 h in Opti-MEM reduced serum media. After 72 h, Opti-MEM was replaced with fresh DMEM, and the cells were grown for an additional 48 h. The cells were treated with trypsin for 5 min, collected by centrifugation at 1000 RPM, and suspended in PBS (1 mL). GFP expression was analyzed by flow cytometry. Data from 10^6^ cells were acquired using a FACS Aria III cell sorter equipped with a 488 nm/blue coherent sapphire solid-state laser, 20 mW (BD Biosciences, San Jose, CA, USA). Data analyses were carried out using FlowJo software (Ashland, OR, USA), according to the manufacturer’s instructions. Parameters, such as MFI and the percentages of specific populations, were quantified by histogram analysis.

Ability of TCO-modified CRISPR suppressors to control nuclease activity was examined as follows: GFP-expressing HEK239 cells were co-transfected with Cas9 mRNA (500 ng, Thermo Fisher Scientific), GFP-targeting **sgRNAs** (30 nM) using lipofectamine (1.5 μL) (Invitrogen™ LMRNA003) for 48 h. Then, **AAA-PNA-TCO, TTT-PNA-TCO, TCO-CPP-RRWQW**, and **TCO-CPP-RLRWR** (10 μM) were added to the media. After 24 h of treatment with the TCO-modified CRISPR suppressors, the media were replaced with fresh DMEM, and the cells were allowed to grow for an additional 48 h. The cells were treated with trypsin for 5 min, collected by centrifugation at 1000 RPM, and suspended in PBS (1 mL). GFP expression was analyzed by flow cytometry. Data from 10^6^ cells were acquired using a FACS Aria III cell sorter equipped with a 488 nm/blue coherent sapphire solid-state laser, 20 mW (BD Biosciences, San Jose, CA, USA). Data analyses were carried out using FlowJo software (Ashland, OR, USA), according to the manufacturer’s instructions. Parameters, such as MFI and the percentages of specific populations, were quantified by histogram analysis.

### Assessment of cell permeability of TCO-modified CRISPR suppressors

The studies were carried out using the previously reported **OG-Tz** fluorescent probe [35]. The chemical structure of **OG-Tz** is shown in Supplementary Fig. S7. **OG-Tz** was synthesized using the procedure reported by Devaraj and co-workers [35]. The HEK293 cells were cultured in DMEM, containing 10% FBS and 1X Penicillin/Streptomycin, at 37°C, 5% CO_2_, and 95% humidity. The cells were seeded at a concentration of 1 × 10^5^ cells per well in a six-well plate 24 h prior to the experiment. The cells were treated with the TCO-modified CRISPR suppressors (30 μM) for 3 h. Then, the media was replaced, and the cells were treated with **OG-Tz** (50 μM) for 2h. Afterwards, the cells were treated with trypsin for 5 min, collected by centrifugation at 1000 RPM, and suspended in PBS (1 mL). GFP expression was analyzed by flow cytometry. Data from 10^6^ cells were acquired using a FACS Aria III cell sorter equipped with a 488 nm/blue coherent sapphire solid-state laser, 20 mW (BD Biosciences, San Jose, CA, USA). Data analyses were carried out using FlowJo software (Ashland, OR, USA), according to the manufacturer’s instructions. Parameters, such as MFI and the percentages of specific populations, were quantified by histogram analysis.

HEK293 cells were grown to ∼70% confluence in 35 mm MatTek glass bottom dishes in DMEM supplemented with 10% bovine serum albumin, penicillin, and streptomycin. The cells were treated with **AAA-PNA-TCO, TTT-PNA-TCO, TCO-CPP-RRWQW**, or **TCO-CPP-RLRWR** (30 μM) for 3 h. Afterwards, the cells were treated with **OG-Tz** (50 μM) for 2 h. Hoechst 33 258 dye was used for nuclear staining. Cellular fluorescence was analyzed using confocal microscopy. Microscopy experiments were carried out using a Zeiss LSM980 confocal microscope.

### Modeling studies

The structure of the Cas9 in complex with RNA and DNA was obtained from the Protein Data Bank (PDB ID: 4OO8 [36]. The solvent accessible surface area (SASA) was computed using the sasa module from GROMACS (Lindahl, Abraham, Hess & van der Spoel Version 2020) employing the default probe radius of 0.14 nm. This radius is representative of water, which is the primary solvent in the system under study. The number and distance of atomic contacts were calculated for the RNA bound to Cas9 using PyMOL (The PyMOL Molecular Graphics System, Version 3.0, Schrödinger, LLC). A cutoff distance of 4Å was used to identify RNA:protein contacts. The Tz1 and Tz2 modifications were introduced on the RNA repeat sequence in MOE (Molecular Operating Environment (MOE), 2024.06 Chemical Computing Group ULC). Local minimization of the structure was then carried out to accommodate the modifications in the complex.

### Modification of sgRNAs with Tz

**Figure 2.**
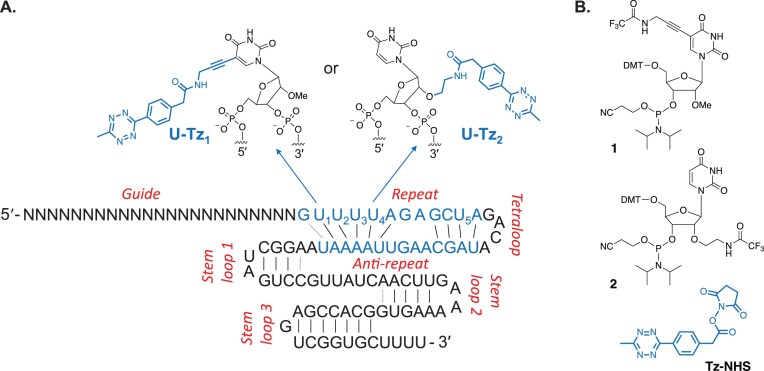
(**A**) Incorporation of Tz into the repeat region of sgRNA. The important uridine residues are numbered U_1_-U_4_. (**B**) Phosphoramidites and **Tz-NHS** ester were used to tag sgRNAs with Tz.

Compound **Tz-NHS** (Fig. 2B) was synthesized using a previously reported method [37]. sgRNA (263 μM) was dissolved in borate buffer (250 μL, pH = 9.4). Compound **Tz-NHS** was dissolved in DMF (400 μL, 50 mM) and added to the RNA. The reaction mixture was vortexed and agitated at 1000 rpm at rt for 8 h. The conjugated sgRNA was purified from small molecules using an Amicon Ultra 3K column (MilliporeSigma, cat# UFC500396) and washed with MQ H_2_O (3 x 300 μL). sgRNAs were purified by preparative PAGE. Purified sgRNAs were analyzed using analytical PAGE, as shown in Supplementary Figs S1 and S2.

### Western blot analysis

Total protein lysate was harvested in RIPA buffer, and proteins separated by 10% SDS-PAGE were transferred to a PVDF membrane by using High MW protocol on Biorad Turbo wet transfer. Following transfer, membranes were blocked in 5% milk in PBST for 1 h at room temperature, washed three times in PBS with 0.1% (w/v) Tween 20 (PBS/T) for 10 min each and placed in primary antibody overnight at 4 °C. Primary antibody was rabbit VEGFA polyclonal antibody (1:2000 dilution, Cat.# 19003–1-AP, Proteintech) and α-tubulin monoclonal antibody (1:20 000 dilution, Cat.# 666031–1-Ig, Proteintech) in 5% BSA in PBS/T. Following overnight incubation, membranes were washed three times in PBS/T for 10 min each. Then membranes were incubated in a 1:10 000 dilution of goat anti-rabbit secondary (Jackson), goat-anti-mouse secondary in 5% milk, and PBS/T. Membranes were washed a final 3 times in PBS/T for 10 min each prior to being imaged on the BioRad ChemiDoc by using Thermo Scientific SuperSignal West Pico PLUS Chemiluminescent Substrate (BioRad, Cat.# 34 577).

## Results and discussion

### Design of constitutively active Tz-tagged sgRNA

We decided to explore the repeat:anti-repeat region of sgRNA for covalent tagging with Tz (Fig. 2A). Several studies showed that it is a highly sensitive and important area of the sgRNA-Cas9 ribonucleoprotein complex. Nishimasu *et al.* reported the crystal structure of Cas9 in complex with sgRNA and target DNA [36]. Cas9 consists of two lobes: a recognition (REC) lobe and a nuclease (NUC) lobe. REC1 and REC2 domains of the REC lobe make direct contacts with the repeat:anti-repeat region of sgRNA. Deletion of either the repeat-interacting region or the anti-repeat-interacting region of the REC1 domain abolished the nuclease activity. Sontheimer *et al.* reported that 2′-OMe RNA modification of all uridines in the repeat:anti-repeat region significantly lowered nuclease activity [34, 38]. Langer and Anderson showed that replacement of the entire repeat:anti-repeat with the corresponding DNA nucleotides completely eliminated nuclease activity [34]. Lastly, Gagnon and Damha reported that replacement of the entire repeat:anti-repeat region with 2′-F-ANA led to an analogous outcome [39].

**Figure 3.**
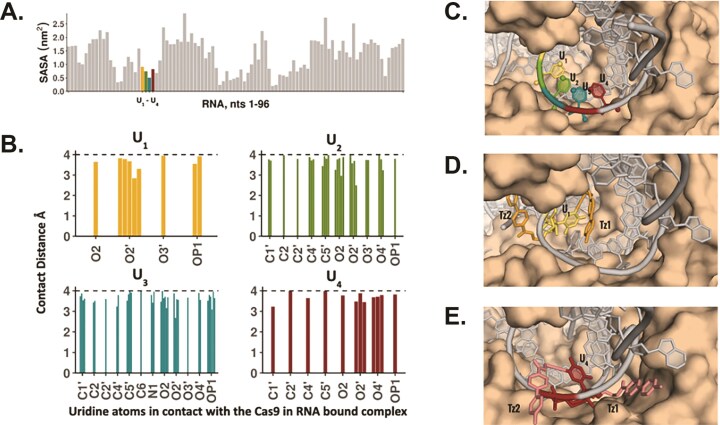
(**A**) Solvent accessible surface area of the RNA nucleotides in the protein:RNA:DNA complex (PDB ID: 4OO8). (**B**) Atomic contacts within 4Å of the four Us of the repeat sequence with Cas9, in the sgRNA–Cas9 complex. (**C**) Structural context of the four Us in the sgRNA–Cas9 complex. The potential modification sites (atoms C5 and O2’) are shown as spheres. sgRNA is shown in gray, and Cas9 is shown in brown. (**D**) Tz_1_ and Tz_2_ modifications modeled on U_1_ (**e**) Tz_1_ and Tz_2_ modifications modeled on U_4_.

To understand the structural context and evaluate the feasibility of tagging the uridines in the repeat sequence of sgRNA with Tz, we carried out molecular modeling studies utilizing the experimentally solved crystal structure of Cas9:RNA/DNA complex [36]. First, the solvent accessible surface area (SASA) of the uridines in the repeat sequence was calculated. This revealed that U_2_ and U_3_ have lower SASA compared to U_1_ and U_4_ (Fig. 3a). U_4_, however, is closer to the peripheral surface of the enzyme compared to U_1_ (Fig. 3c). Furthermore, the number of contacts with Cas9, defined as any atoms of the Us within 4Å of the enzyme, is lower for U_1_ and U_4_ compared to U_2_ and U_3_ (Fig. 3b). While C5, the site for the Tz_1_ modification, has no contacts with the enzyme, the number of contacts for 2′-O, the site for the Tz_2_ modification, is higher in the case of U_1_. Additionally, modeling the Tz_1_ and Tz_2_ modifications on both U_1_ and U_4_ revealed that the modifications can be tolerated with minor local rearrangements and minimal disruption of the RNA:enzyme interface in the REC1 domain (Fig. 3d & 3e). This is particularly true for U_4_, where the modified groups point away from the complex. Thus, the modeling predicted that while sgRNA modified with Tz_1_ or Tz_2_ at U_1_ and U_4_ should remain active, the modifications might be more accessible for accommodating additional chemistries at position U_4_.

Following these predictions, we synthesized four sgRNAs targeting the linearized pBR322 plasmid by solid-phase RNA synthesis. The modified uridine phosphoramidites **1** and **2** (Fig. 2B) were incorporated into positions U_1_ or U_4_. Chemical synthesis of these phosphoramidites has previously been reported [37]. Tz was tagged post-synthetically using **Tz-NHS** (Fig. 2B). We termed the constructs **sgRNA1-(U_1_Tz_1_), sgRNA1-(U_1_Tz_2_), sgRNA1-(U_4_Tz_1_)**, and **sgRNA1-(U_4_Tz_2_)** to indicate the type of Tz modification and the position where it was installed. Following the synthesis, the four Tz-tagged sgRNAs were purified by preparative PAGE. Successful synthesis and purification were confirmed by denaturing gel electrophoresis (Supplementary Fig. S1). Selected constructs were also characterized by ESI-MS (Supplementary Fig. S1).

We tested the ability of different sgRNAs to cut the linearized pBR322 plasmid in the presence of Cas9. We determined that 16 h of treatment was optimal for quantitative comparison between different sgRNA variants. Supplementary Fig. S10 shows agarose gel electrophoresis analysis of nuclease activity at 1, 2, 4, and 16 h timepoints using the unmodified **sgRNA1**. We observed an incremental increase in the amount of cut DNA as a function of time. Subsequently, we tested the nuclease activity of different sgRNA variants at 1 and 16 h timepoints (Supplementary Fig. S11). **sgRNA1-(U_1_Tz_2_)** showed no detectable activity after 1 h, thus suggesting that it’s completely inactive. However, the same construct showed 40% activity after 16 h. Meanwhile, **sgRNA1-(U_2_Tz_2_)** and **sgRNA1-(U_3_Tz_2_)** did not have any detectible activity after 1 or 16 h of incubation, thus indicating that these constructs are indeed completely inactive. Therefore, all subsequent quantitative comparisons of *in vitro* CRISPR-Cas9 experiments were done after 16 h of treatment.

**Figure 4.**
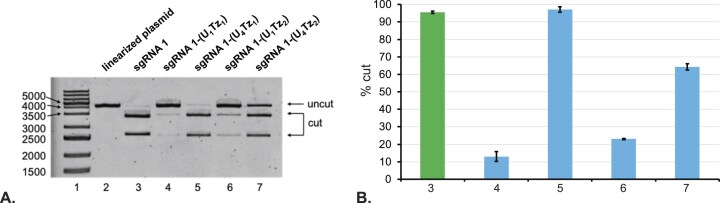
Analysis of Cas9-enabled nuclease activity in the presence of different Tz-tagged sgRNAs using agarose gel electrophoresis. The linearized pBR322 plasmid was treated with Cas9 and different Tz-tagged sgRNAs for 16 h. Unmodified **sgRNA1** was used as a positive control. (**A**) *Lane 1*: DNA ladder; *Lane 2*: linearized pBR322 plasmid; *Lane 3*: linearized pBR322 plasmid treated with Cas9 and **sgRNA1**; *Lane 4*: linearized pBR322 plasmid treated with Cas9 and **sgRNA1-(U_1_Tz_1_)**; *Lane 5*: linearized pBR322 plasmid treated with Cas9 and **sgRNA1-(U_4_Tz_1_)**; *Lane 6*: linearized pBR322 plasmid treated with Cas9 and **sgRNA1-(U_1_Tz_2_)**; *Lane 7*: linearized pBR322 plasmid treated with Cas9 and **sgRNA1-(U_4_Tz_2_)**. (**B**) Bar chart representing the percentage of cut DNA upon treatment with the constructs in part **A**. Lane numbering is the same as in part **A**. All CRISPR experiments were performed in duplicate. Error bars represent ± S.D.

Fig. 4 illustrates agarose gel electrophoresis analysis of CRISPR-Cas9 experiments that were carried out for 16 h. **sgRNA1-(U_4_Tz_1_)**, shown in lane 5, was found to have the same level of activity as the unmodified **sgRNA1. sgRNA1-(U_4_Tz_2_)**, shown in lane 7, was also found to have strong activity that was slightly lower than the unmodified **sgRNA1**. Meanwhile, modification at the U_1_-position with Tz_1_, shown in lane 4, resulted in a 7.3-fold decrease of nuclease activity. Analogously, modification at the U_1_-position with Tz_2_, shown in lane 6, resulted in a 4.2-fold decrease of nuclease activity. These results suggested that constitutively functional sgRNA, described in Fig. 1, can be achieved by tagging the U_4_-position with either Tz_1_ or Tz_2_.

**Figure 5.**
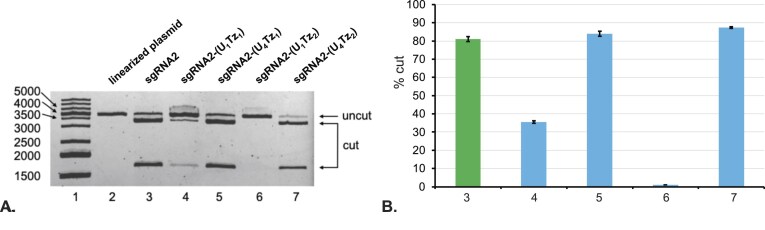
Analysis of Cas9-enabled nuclease activity in the presence of different Tz-tagged sgRNAs using agarose gel electrophoresis. Linearized eGFP-N1 plasmid was treated with Cas9 and different Tz-tagged sgRNAs for 16 h. Unmodified **sgRNA2** was used as a positive control. (**A**) *Lane 1*: DNA ladder; *Lane 2*: linearized eGFP-N1 plasmid; *Lane 3*: linearized eGFP-N1 plasmid treated with Cas9 and **sgRNA2**; *Lane 4*: linearized eGFP-N1 plasmid treated with Cas9 and **sgRNA2-(U_1_Tz_1_)**; *Lane 5*: linearized eGFP-N1 plasmid treated with Cas9 and **sgRNA2-(U_4_Tz_1_)**; *Lane 6*: linearized eGFP-N1 plasmid treated with Cas9 and **sgRNA2-(U_1_Tz_2_)**; *Lane 7*: linearized eGFP-N1 plasmid treated with Cas9 and **sgRNA2-(U_4_Tz_2_)**. (**B**) Bar chart representing the percentage of cut DNA upon treatment with the constructs in part **A**. Lane numbering is the same as in part **A**. All CRISPR experiments were performed in duplicate. Error bars represent ± s.d.

To test the generality of our tagging approach, we synthesized sgRNAs targeting a completely different, linearized eGFP-N1 plasmid. We analogously termed them **sgRNA2-(U_1_Tz_1_), sgRNA2-(U_1_Tz_2_), sgRNA2-(U_4_Tz_1_)** and **sgRNA2-(U_4_Tz_2_)**. Following the synthesis, the four Tz-tagged sgRNAs were purified by preparative PAGE (Supplementary Fig. S2) and subsequently tested for their ability to cut linearized eGFP-N1 plasmid in the presence of Cas9. After 16 h of treatment, the experiments were analyzed by agarose gel electrophoresis, shown in Fig. 5. Once again, we observed that tagging of U_4_ with either Tz_1_ or Tz_2_ conserved the nuclease activity (lanes 5 and 7). Meanwhile, modification at the U_1_-position with Tz_1_, shown in lane 4, resulted in a 2.3 reduction of nuclease activity. Modification of the same position with Tz_2_ (lane 6) completely inhibited nuclease activity. The data indicated a general principle that sgRNAs tagged with either Tz_1_ or Tz_2_ at the U_4_ position of the repeat region will be constitutively active.

### Design and synthesis of TCO-CRISPR suppressors

**Figure 6.**
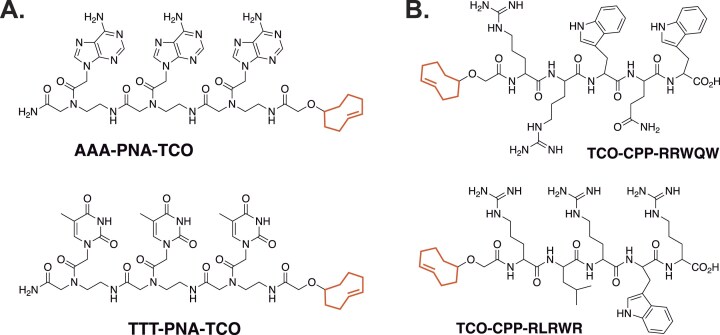
A library of small molecule TCO-modified CRISPR suppressors that are based on (**A**.) peptide nucleic acids (PNAs); (**B**.) small cell-penetrating peptides (CPPs).

After achieving constitutively functional, Tz-tagged sgRNA, we synthesized four TCO-modified CRISPR suppressors, shown in Fig. 6. The compounds have been designed to be modular small molecules with molecular weights under 1200 Da. This will allow for facile TCO modification and optimization of functional groups that will interfere with the structural elements of the sgRNA–Cas9 complex. The first set of suppressor compounds (Fig. 6A) is peptide nucleic acids (PNAs) containing either thymine or adenine nucleobases. We hypothesize that either thymine or adenine nucleobases of the PNAs will interfere with the native repeat-anti-repeat structure that contains five U-A base pairs. Because PNAs can be synthesized in a programmable manner by solid-phase synthesis, they can be further optimized in terms of length and sequence to find the optimal disrupter of the sgRNA–Cas9 complex. The second set of compounds is TCO-CPP constructs (Fig. 6B) that are based on the smallest-known cell-penetrating peptides (CPPs) [40–42]. They are also small molecules with an MW under 1200 Da. As the name implies, they have been shown to be cell-permeable and capable of shuttling various therapeutic cargoes across the cellular membrane. These short peptides can be easily synthesized by standard solid-phase peptide synthesis. Post-synthetically, they were modified with TCO and purified by HPLC. In addition to inherent steric hindrance, guanidinium groups of the shown TCO-CPP constructs were expected to interfere with nucleobase base-pairing in the repeat:anti-repeat region of sgRNA.

**Figure 7.**
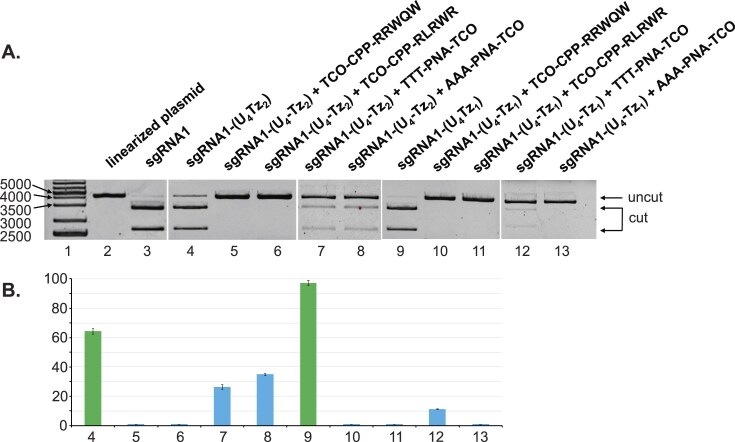
Analysis of the impact of different TCO-modified CRISPR suppressors on Cas9-enabled nuclease activity using agarose gel electrophoresis. Linearized pBR322 plasmid was treated with Cas9, either **sgRNA1-(U_4_Tz_1_)** or **sgRNA1-(U_4_Tz_2_)**, and different TCO-modified CRISPR suppressors for 16 h. (**A**) *Lane 1*: DNA ladder; *Lane 2*: linearized pBR322 plasmid; *Lane 3*: linearized pBR322 plasmid treated with Cas9 and **sgRNA1**; *Lane 4*: linearized pBR322 plasmid treated with Cas9 and **sgRNA1-(U_4_Tz_2_)**; *Lane 5*: linearized pBR322 plasmid treated with Cas9**, sgRNA1-(U_4_Tz_2_)** and **TCO-CPP-RRWQW**; *Lane 6*: linearized pBR322 plasmid treated with Cas9, **sgRNA1-(U_4_Tz_2_)** and **TCO-CPP-RLRWR**; *Lane 7*: linearized pBR322 plasmid treated with Cas9, **sgRNA1-(U_4_Tz_2_)** and **TTT-PNA-TCO**; *Lane 8*: linearized pBR322 plasmid treated with Cas9, **sgRNA1-(U_4_Tz_2_)** and **AAA-PNA-TCO**; *Lane 9*: linearized pBR322 plasmid treated with Cas9 and **sgRNA1-(U_4_Tz_1_)**; *Lane 10*: linearized pBR322 plasmid treated with Cas9, **sgRNA1-(U_4_Tz_1_)** and **TCO-CPP-RRWQW**; *Lane 11*: linearized pBR322 plasmid treated with Cas9, **sgRNA1-(U_4_Tz_1_)** and **TCO-CPP-RLRWR**; *Lane 12*: linearized pBR322 plasmid treated with Cas9, **sgRNA1-(U_4_Tz_1_)** and **TTT-PNA-TCO**; *Lane 13*: linearized pBR322 plasmid treated with Cas9, **sgRNA1-(U_4_Tz_1_)** and **AAA-PNA-TCO**. (**B**) Bar chart representing the percentage of cut DNA upon treatment with the constructs in part **A**. Lane numbering is the same as in part **A**. All CRISPR experiments were performed in duplicate. Error bars represent ± S.D.

Upon completion of their synthesis, the TCO-modified CRISPR suppressors were assessed for their ability to control the Cas9-assisted nuclease activity of **sgRNA1-(U_4_Tz_1_)** and **sgRNA1-(U_4_Tz_2_)**. The linearized pBR322 plasmid was treated with Cas9 and either **sgRNA1-(U_4_Tz_1_)** or **sgRNA1-(U_4_Tz_2_)**. In parallel, the same experiments were done in the presence of the TCO-modified CRISPR suppressors. After 16 h of treatment, the experiments were analyzed by agarose gel electrophoresis, as shown in Fig. 7A. The gel data were quantified and plotted as a bar graph in Fig. 7B. The CPP-based ligands showed the most promising ability to suppress nuclease activity. **TCO-CPP-RRWQW** and **TCO-CPP-RLRWR** completely inhibited activity of **sgRNA1-(U_4_Tz_2_)** (lanes 5 and 6) and **sgRNA1-(U_4_Tz_1_)** (lanes 10 and 11). Meanwhile, the PNA-based suppressors did not prove to be as effective. **sgRNA1-(U_4_Tz_2_)** treated with **TTT-PNA-TCO** or **AAA-PNA-TCO** still retained some residual nuclease activity (26% and 35%).

**Figure 8.**
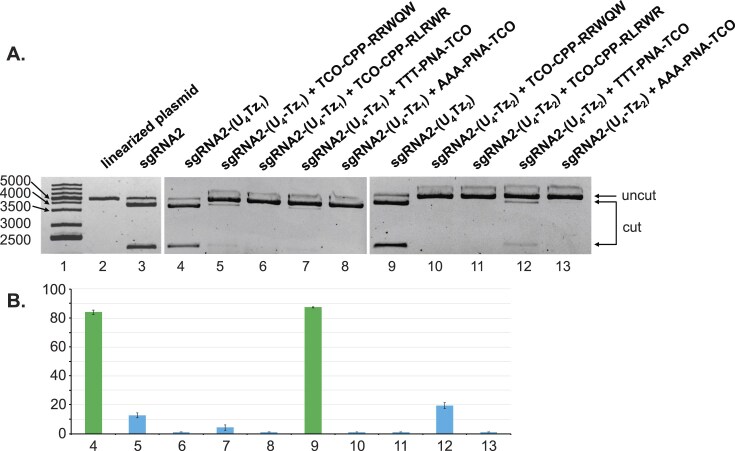
Analysis of the impact of different TCO-modified CRISPR suppressors on Cas9-enabled nuclease activity using agarose gel electrophoresis. Linearized eGFP-N1 plasmid was treated with Cas9, either **sgRNA1-(U_4_Tz_1_)** or **sgRNA1-(U_4_Tz_2_)**, and different TCO-modified CRISPR suppressors for 16 h. (**A**) *Lane 1*: DNA ladder; *Lane 2*: linearized eGFP-N1 plasmid; *Lane 3*: linearized eGFP-N1 plasmid treated with Cas9 and **sgRNA2**; *Lane 4*: linearized eGFP-N1 plasmid treated with Cas9 and **sgRNA2-(U_4_Tz_1_)**; *Lane 5*: linearized eGFP-N1 plasmid treated with Cas9, **sgRNA2-(U_4_Tz_1_)** and **TCO-CPP-RRWQW**; *Lane 6*: linearized eGFP-N1 plasmid treated with Cas9, **sgRNA2-(U_4_Tz_1_)** and **TCO-CPP-RLRWR**; *Lane 7*: linearized eGFP-N1 plasmid treated with Cas9, **sgRNA2-(U_4_Tz_1_)** and **TTT-PNA-TCO**; *Lane 8*: linearized eGFP-N1 plasmid treated with Cas9, **sgRNA2-(U_4_Tz_1_)** and **AAA-PNA-TCO**; *Lane 9*: linearized eGFP-N1 plasmid treated with Cas9 and **sgRNA2-(U_4_Tz_2_)**; *Lane 10*: linearized eGFP-N1 plasmid treated with Cas9, **sgRNA2-(U_4_Tz_2_)** and **TCO-CPP-RRWQW**; *Lane 11*: linearized eGFP-N1 plasmid treated with Cas9, **sgRNA2-(U_4_Tz_2_)** and **TCO-CPP-RLRWR**; *Lane 12*: linearized eGFP-N1 plasmid treated with Cas9, **sgRNA2-(U_4_Tz_2_)** and **TTT-PNA-TCO**; *Lane 13*: linearized eGFP-N1 plasmid treated with Cas9, **sgRNA2-(U_4_Tz_2_)** and **AAA-PNA-TCO**. (**B**) Bar chart representing the percentage of cut DNA upon treatment with the constructs in part **A**. Lane numbering is the same as in part **A**. All CRISPR experiments were performed in duplicate. Error bars represent ± S.D.

To test the generality of our CRISPR-Cas9 regulation approach, we performed analogous experiments using **sgRNA2-(U_4_Tz_1_)** and **sgRNA2-(U_4_Tz_2_)** targeting the linearized eGFP-N1 plasmid. After 16 h of treatment, the experiments were analyzed by agarose gel electrophoresis, as shown in Fig. 8A. The gel data were quantified and plotted as a bar graph in Fig. 8B. In the case of **sgRNA2-(U_4_Tz_2_), TCO-CPP-RRWQW** (lane 5) and **TTT-PNA-TCO** (lane 7) addition resulted in a residual nuclease activity of 13% and 4%, respectively. Addition of **TCO-CPP-RLRWR** and **AAA-PNA-TCO** completely inhibited nuclease activity (lanes 6 and 8). In the case of **sgRNA2-(U_4_Tz_1_), TTT-PNA-TCO** (lane 12) partially lowered nuclease activity to 20%. Meanwhile, the addition of **TCO-CPP-RRWQW, TCO-CPP-RLRWR**, and **AAA-PNA-TCO** completely inhibited nuclease activity (lanes 10, 11, and 13). In a control experiment, we tested the impact of TCO-modified CRISPR suppressors on the activity of the unmodified **sgRNA2** (Supplementary Fig. S12). We found that their addition had little effect on the nuclease associated with **sgRNA2**. This suggests that the TCO-modified CRISPR suppressors inhibit nuclease activity only upon their click reaction with the Tz-tagged sgRNAs. In conclusion, the *in vitro* data suggest that the paradigm, schematically shown in Fig. 1, can be achieved using sgRNA modified with either Tz_1_ or Tz_2_ at the U_4_ position of the repeat region. Inactivation of Tz-tagged sgRNAs can be best achieved using either **TCO-CPP-RRWQW** or **TCO-CPP-RLRWR**.

### Cell studies

**Figure 9.**
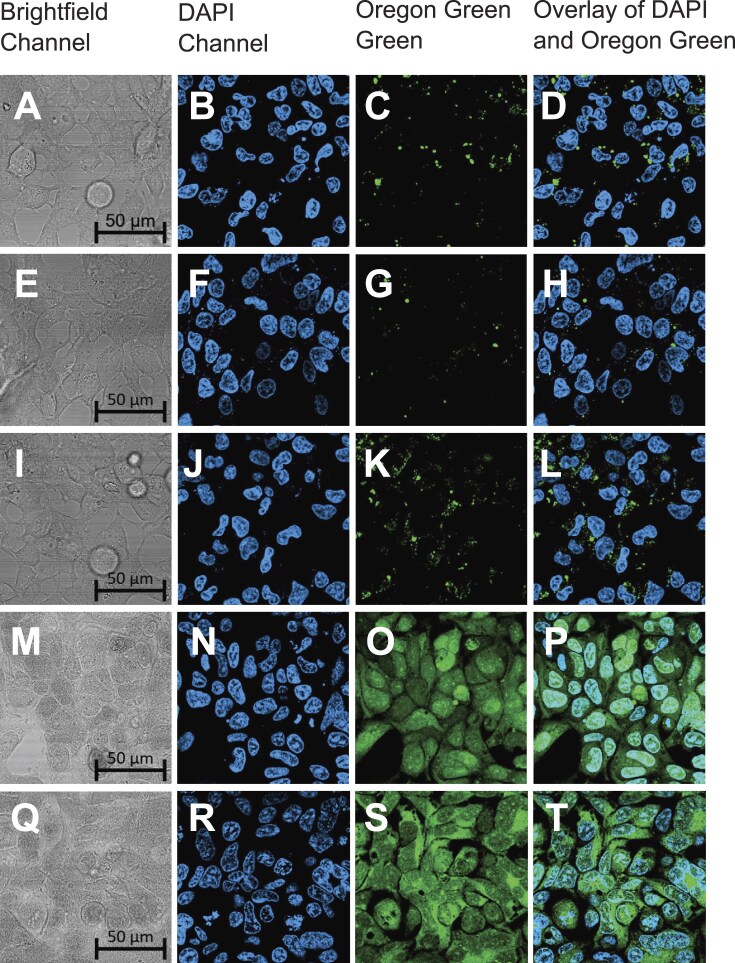
Assessment of cell permeability of TCO-modified CRISPR suppressors. HEK293 cells were treated with **OG-Tz** alone (**A**-**D**), or in combination with **AAA-PNA-TCO** (**E**-**H**), **TTT-PNA-TCO** (**I**-**L**), **TCO-CPP-RRWQW** (**M**-**P**), and **TCO-CPP-RLRWR** (**Q**-**T**).

We assessed cell permeability of the TCO-modified CRISPR suppressors using the **OG-Tz** fluorescent probe, shown in Supplementary Fig. S7. **OG-Tz** is a π-conjugated alkenyl tetrazine, reported by Devaraj and co-workers [35]. It’s been shown to quench fluorescence of the Oregon Green dye via through-bond energy transfer (TBET) mechanism [43]. The fluorescence signal can be restored upon the click reaction with TCO. We tested the fluorescence response of **OG-Tz** upon addition of the TCO-modified CRISPR suppressors in PBS (pH 7.4). As illustrated in Supplementary Fig. S7, there is a 10-fold enhancement of fluorescence. The cells were treated with **AAA-PNA-TCO, TTT-PNA-TCO, TCO-CPP-RRWQW**, or **TCO-CPP-RLRWR** for 3 h. Afterwards, the cells were treated with **OG-Tz** (50 μM) for 2 h. Hoechst 33 258 dye was used for nuclear staining. Cellular fluorescence was analyzed using microscopy and flow cytometry. The microscopy data is shown in Fig. 9. As expected, the cells treated with **OG-Tz** alone showed weak fluorescence in the Oregon Green channel (Fig. 9C). Punctate staining was observed in cells treated with **OG-Tz** and **AAA-PNA-TCO** and **TTT-PNA-TCO** (Fig. 9G and K). The strongest fluorescence was observed in cells treated with **OG-Tz** and either **TCO-CPP-RRWQW** or **TCO-CPP-RLRWR** (Fig. 9O and S), suggesting that the CPP compounds have good cell permeability. The cells shown in Fig. 9 were trypsinized and analyzed by flow cytometry, which confirmed the findings (Supplementary Fig. S8).

Control of CRISPR-Cas9 activity was assessed in GFP-expressing HEK239 cells. We were concerned about the post-transfection stability of **sgRNA2** that might be exposed to nuclease degradation inside the cells. This concern was addressed by site-specific modification of **sgRNA2** with 2′-OMe groups. We followed the strategy described by Yin *et al.*, who identified the exact positions that can be modified with 2′-OMe without significant perturbation to native binding between sgRNA and Cas9 [44]. We synthesized **sgRNA3**, having the sequence: 5′-GGGCGAGGAGCUGUUC ACCGGU_1_U_2_U_3_U_4_AGagcuagaaauagcaaGUUaA aAuAaggcuaGUccGUUAucAAcuugaaaaagugGcaccgagucggugcuuuuU-3′. Capital letters indicate unmodified nucleotides, while small letters correspond to nucleotides containing 2′-OMe groups. For reference, uridines of the repeat region are numbered 1–4. U_4_ was tagged with either Tz_1_ or Tz_2_, thus making the constructs **sgRNA3-(U_4_Tz_1_)** and **sgRNA3-(U_4_Tz_2_)**. We assessed the CRISPR-Cas9 activity of these constructs *in vitro* (Supplementary Figs S9). The *in vitro* experiments showed that 2′-OMe modifications did not perturb nuclease activity, and the new constructs behave similarly to **sgRNA2-(U_4_Tz_1_)** and **sgRNA2-(U_4_Tz_2_)**.

**Figure 10.**
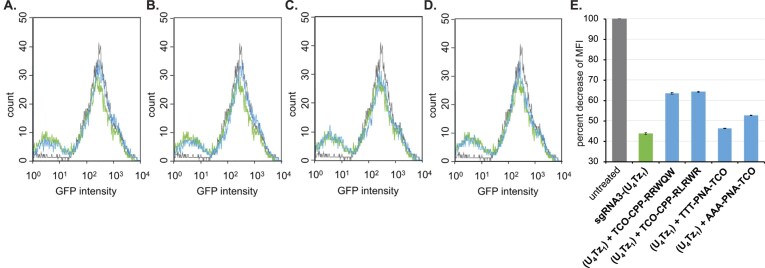
Analysis of CRISPR-Cas9 activity after addition of TCO-modified CRISPR suppressors using flow cytometry. In one experiment, GFP-expressing HEK293 cells were transfected with Cas9 mRNA and **sgRNA 3-(U_4_Tz_1_)** for 72 h. In parallel, the cells were transfected with Cas9 mRNA and **sgRNA 3-(U_4_Tz_1_)** for 48 h, followed by treatment with different TCO-modified CRISPR suppressors for 24 h. The flow cytometry data are represented as an overlay of histograms of the untreated cells (black), cells transfected with Cas9 and **sgRNA 3-(U_4_Tz_1_)** (green), and cells subsequently treated with (**A**) **TCO-CPP-RRWQW**; (**B**) **TCO-CPP-RLRWR**; (**C**) **TTT-PNA-TCO**; (**D**) **AAA-PNA-TCO** (blue); (**E**) decrease of MFI of GFP relative to the untreated GFP-expressing HEK293 cells. All experiments were performed in duplicate. Error bars represent ± s.d.

**Figure 11.**
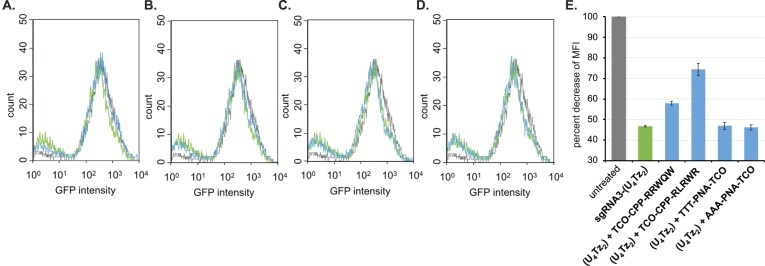
Analysis of CRISPR-Cas9 activity after addition of TCO-modified CRISPR suppressors using flow cytometry. In one experiment, GFP-expressing HEK293 cells were transfected with Cas9 mRNA and **sgRNA 3-(U_4_Tz_2_)** for 72 h. In parallel, the cells were transfected with Cas9 mRNA and **sgRNA 3-(U_4_Tz_2_)** for 48 h, followed by treatment with different TCO-modified CRISPR suppressors for 24 h. The flow cytometry data are represented as an overlay of histograms of the untreated cells (black), cells transfected with Cas9 and **sgRNA 3-(U_4_Tz_2_)** (green), and cells subsequently treated with (**A**) **TCO-CPP-RRWQW**; (**B**) **TCO-CPP-RLRWR**; (**C**) **TTT-PNA-TCO**; (**D**) **AAA-PNA-TCO** (blue); (**E**) decrease of MFI of GFP relative to the untreated GFP-expressing HEK293 cells. All experiments were performed in duplicate. Error bars represent ± S.D.

GFP-expressing HEK239 cells were co-transfected with **sgRNA3, sgRNA3-(U_4_Tz_1_)**, or **sgRNA3-(U_4_Tz_2_)** and commercially available mRNA that encodes the Cas9 gene for 72 h. After the transfection, the media were replaced with fresh DMEM, and the cells were allowed to grow for an additional 48 h. GFP expression was analyzed by flow cytometry and compared to the untreated cells (Supplementary Fig. S13, Figs10 and11). The Tz-tagged sgRNAs were found to be as effective at targeting the GFP gene as **sgRNA3** (Supplementary Fig. S13). As illustrated in Figs. 10E, the mean fluorescence intensity (MFI) of GFP decreased by 44% after **sgRNA3-(U_4_Tz_1_)** treatment. Similarly, MFI of GFP decreased by 47% after **sgRNA3-(U_4_Tz_2_)** treatment (Fig. 11E). The ability of TCO-modified CRISPR suppressors to control nuclease activity was examined in the next set of experiments. GFP-expressing HEK239 cells were co-transfected with **sgRNA3-(U_4_Tz_1_)** or **sgRNA3-(U_4_Tz_2_)** and commercially available mRNA that encodes the Cas9 gene for 48 h. Then, **AAA-PNA-TCO, TTT-PNA-TCO, TCO-CPP-RRWQW**, and **TCO-CPP-RLRWR** (10 μM) were added to the media. After 24 h of treatment with the TCO-modified CRISPR suppressors, the media were replaced with fresh DMEM, and the cells were allowed to grow for an additional 48 h. As illustrated in Figs 10A, E and 11A, E, **TCO-CPP-RRWQW** was able to inactivate **sgRNA3-(U_4_Tz_1_)** and **sgRNA3-(U_4_Tz_2_)** inside the cells. In these experiments, the MFI of GFP decreased by lower amounts as a result of turning off the CRISPR–Cas9 complex. Analogous results were observed after treatment with **TCO-CPP-RLRWR** (Figs. 10B, 10E, 11B and 11E). However, the PNA-based CRISPR suppressors were not as effective, as shown in Figs 10C, D, E and11C, D, E. The observed MFI of GFP was very close to that of the cells treated with **sgRNA3-(U_4_Tz_1_)** or **sgRNA3-(U_4_Tz_2_)** alone. This is probably due to inferior cell permeability of **AAA-PNA-TCO** and **TTT-PNA-TCO**, as was observed in Fig. 9.

To illustrate the therapeutic potential of our technology, we implemented it towards a well-established medicinal target, vascular endothelial growth factor A (VEGFA). VEGFA is an angiogenic factor, whose expression is upregulated in neovascular age-related macular degeneration, the leading cause of vision loss [45, 46]. CRISPR-Cas9-based disruption of VEGFA has already been explored as a therapeutic strategy [47, 48]. We designed **sgRNA4-(U_4_Tz_1_)** and **sgRNA4-(U_4_Tz_2_)**, where the guide region was programmed to target the VEGFA gene.

**Figure 12.**
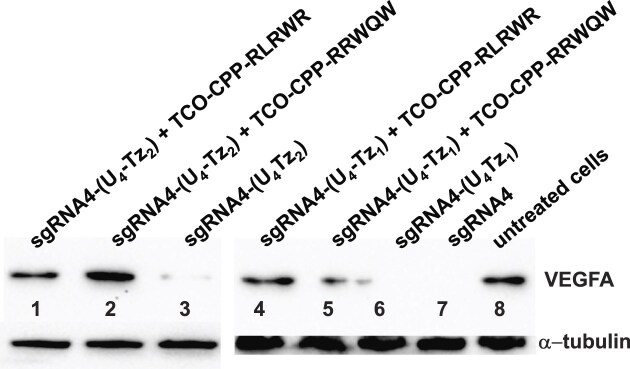
Western blot analysis of CRISPR-Cas9 experiments targeting VEGFA. HEK293T cells were transfected with Cas9 mRNA and either **sgRNA 4-(U_4_Tz_1_)** or **sgRNA 4-(U_4_Tz_2_)** for 72 h. In parallel, the cells were transfected with the same compounds for 48 h, followed by treatment with different TCO-modified CRISPR suppressors for 24 h. Cells were treated with: *Lane 1*: **sgRNA4-(U_4_Tz_2_)** and **TCO-CPP-RLRWR**; *Lane 2*: **sgRNA4-(U_4_Tz_2_)** and **TCO-CPP-RRWQW**; *Lane 3*: **sgRNA4-(U_4_Tz_2_)**; *Lane 4*: linearized eGFP-N1 plasmid**, sgRNA2-(U_4_Tz_1_)** and **TCO-CPP-RLRWR**; *Lane 5*: **sgRNA4-(U_4_Tz_1_)** and **TCO-CPP-RRWQW**; *Lane 6*: **sgRNA4-(U_4_Tz_1_)**; *Lane 7*: **sgRNA4**; *Lane 8*: untreated cells. α-tubulin was used as a loading control.

To assess the new constructs, HEK239T cells were co-transfected with the unmodified **sgRNA4, sgRNA4-(U_4_Tz_1_)**, or **sgRNA4-(U_4_Tz_2_)** and commercially available mRNA that encodes the Cas9 gene for 72 h. After the transfection, the media were replaced with fresh DMEM, and the cells were allowed to grow for an additional 48 h. VEGFA expression was analyzed by Western blot, as shown in Fig. 12. We observed a marked decrease in VEGFA expression in sgRNA-Cas9-treated cells. The Tz-tagged sgRNAs had similar levels of VEGFA suppression as the unmodified **sgRNA4**. We repeated these experiments, while adding **TCO-CPP-RRWQW** and **TCO-CPP-RLRWR** (10 μM) after 48 hours of transfection. VEGFA expression was once again analyzed by Western blot. Addition of TCO-modified CRISPR suppressors resulted in higher VEGFA expression, suggesting that Tz-tagged sgRNAs were inactivated inside the cells.

## Conclusion

Here, we describe a bio-orthogonal-chemistry-based methodology to switch off CRISPR–Cas9 nuclease activity. We anticipate that this approach will be particularly useful for CRISPR drug-delivery strategies that can prolong cellular exposure to active gene editing components, thereby increasing the risk of undesirable off-target editing. For example, LNP formulations that co-encapsulate heavily modified sgRNA together with Cas9-encoding mRNA may sustain Cas9 expression and extend the effective lifetime of the active ribonucleoprotein complex. In this context, our technology enables nuclease inactivation after the desired on-target edit has been achieved, potentially limiting continued editing at off-target sites.

While developing our methodology, we identified a precise position within the repeat:anti-repeat region of sgRNA that can be tagged with Tz without disrupting Cas9-enabled nuclease activity. This is known to be a highly sensitive region of sgRNA, and we chose it for tagging to enable regulation of RNA-protein interactions. We carried out Tz-tagging of three different sgRNAs to illustrate that this strategy is general. Afterwards, we designed TCO-modified small-molecule CRISPR suppressors that can be assembled in a modular fashion and that, upon clicking to Tz, significantly reduce the system’s nuclease activity. Of the tested molecules, CPP-based suppressors showed the most effective reduction of CRISPR activity both in solution and live cells. Overall, the described strategy to control CRISPR-Cas9 nuclease activity consists of a single small-molecule tag and a small-molecule cell-permeable suppressor. In comparison to the previously reported systems to control CRISPR-Cas9 nuclease activity, our design entails minimal perturbation to the native sgRNA-Cas9 complex. Lastly, we illustrated the therapeutic potential of our technology by targeting the VEGFA gene in live HEK293T cells. In the future, we plan to examine if our technology can reduce off-target effects using the GUIDE-Seq protocol [13]. We also plan to expand the library of TCO-modified small-molecule CRISPR suppressors to investigate the precise mechanism of inactivation of nuclease activity.

## Supplementary Material

ugag008_Supplemental_File

## Data Availability

The data underlying this article are available in the article and in its online supplementary material.
