# Supplementary material for: Bio-orthogonal chemistry-based strategy to Turn-OFF CRISPR-Cas9 activity in solution and live cells
Source: NAR Mol Med. 2026 Jan 30;3(1):ugag008. doi: 10.1093/narmme/ugag008 (PMC12891993; doi:10.1093/narmme/ugag008)
Supplement: ugag008_Supplemental_File [file ugag008_supplemental_file.pdf]

SUPPORTING INFORMATION

Title: Bio-Orthogonal Chemistry-Based Strategy to Turn-OFF CRISPR-Cas9 Activity in Solution and Live Cells.

Authors: Bhoomika Pandit[1], Sweta Vangaveti[2], Justa Fidelity Sentre[1], Ian McClain[1] Gabriele Fuchs[3], Maksim Royzen[1]*

Affiliations:

[1] Department of Chemistry, University at Albany, 1400 Washinton Ave. Albany, NY 12208
[2] The RNA Institute, University at Albany, 1400 Washinton Ave. Albany, NY 12208
[3] Department of Biology, University at Albany, 1400 Washinton Ave. Albany, NY 12208

* email: mroyzen@albany.edu

Table of Contents	Figure and Scheme titles	Page
RNA Sequences		S2-S3
Figure S1	PAGE analysis of purified sgRNAs	S4
Figure S2	PAGE analysis of purified sgRNAs	S4
Figure S3	Analytical HPLC and ESI-MS spectra of **TCO-CPP-RRWQW**	S5
Figure S4	Analytical HPLC and ESI-MS spectra of **TCO-CPP-RLRWR**	S6
Figure S5	Analytical HPLC and ESI-MS spectra of **AAA-PNA-TCO**	S7
Figure S6	Analytical HPLC and ESI-MS spectra of **TTT-PNA-TCO**	S8
Figure S7	Fluorescence spectra of **OG-Tz** conjugated to different TCO-modified CRISPR suppressors	S9
Figure S8	Flow cytometry of HEK293 cells treated with **OG-Tz** alone and with **OG-Tz** and TCO-modified CRISPR suppressors	S9
Figure S9	Analysis of CRISPR-Cas9 experiments using agarose gel electrophoresis	S10
Figure S10	Optimization of Cas9-enabled nuclease activity in the presence of unmodified **sgRNA1** using agarose gel electrophoresis.	S10
Figure S11	Optimization of CRISPR-Cas9 experiments	S10
Figure S12	Analysis of impact of different TCO-modified CRISPR suppressors on Cas9-enabled nuclease activity of **sgRNA2**	S11
Figure S13	Flow cytometry experiments that compare in-cell nuclease activity of **sgRNA 3, sgRNA 3-(U_4Tz_1)** and **sgRNA 3-(U_4Tz_2)**	S12

RNA Sequences:

pBR322-targeting sgRNA:

sgRNA1-(U_1Tz_1)
5′ - GGGCGCUUGUUUCGGCGUGGGUAG**U_1-Tz1$U_2U_3U_4$**AGAGCUAGACAUAGC
AAGUUAAAAUAAGGCUAGUCCGUUAUCAACUUGAAAAAGUGGCACCGAGUCGGU
GCUUUU - 3′

sgRNA1-(U_4Tz_1)
5′ -GGGCGCUUGUUUCGGCGUGGGUAG**U_1U_2U3U_4-Tz1**AGAGCUAGACAUAGC
AAGUUAAAAUAAGGCUAGUCCGUUAUCAACUUGAAAAAGUGGCACCGAGUCGGU
GCUUUU - 3′

sgRNA1-(U_1Tz_2)
5′ - GGGCGCUUGUUUCGGCGUGGGUAG**U_1-Tz2$U_2U_3U_4$**AGAGCUAGACAUAGC
AAGUUAAAAUAAGGCUAGUCCGUUAUCAACUUGAAAAAGUGGCACCGAGUCGGU
GCUUUU - 3′

sgRNA1-(U_4Tz_2):
5′ -GGGCGCUUGUUUCGGCGUGGGUAG**U_1U_2U3U_4-Tz2**AGAGCUAGACAUAGC
AAGUUAAAAUAAGGCUAGUCCGUUAUCAACUUGAAAAAGUGGCACCGAGUCGGU
GCUUUU - 3′

eGFP-targeting sgRNA:

sgRNA2-(U_1Tz_1)
5’-GGGCGAGGAGCUGUUCACCGG**U_1-Tz1$U_2U_3U_4$**AGAGCUAGAAAUAGCAAGUU
AAAAUAAGGCUAGUCCGUUAUCAACUUGAAAAAGUGGCACCGAGUCGGUGCUUU
UU-3’

sgRNA2-(U_4Tz_1)
5’-GGGCGAGGAGCUGUUCACCGG**$U_1U_2U_3U_4$-Tz1**AGAGCUAGAAAUAGCAAGUU
AAAAUAAGGCUAGUCCGUUAUCAACUUGAAAAAGUGGCACCGAGUCGGUGCUUU
UU-3’

sgRNA2-(U_1Tz_2)
5’-GGGCGAGGAGCUGUUCACCGG**U_1-Tz2$U_2U_3U_4$**AGAGCUAGAAAUAGCAAGUU
AAAAUAAGGCUAGUCCGUUAUCAACUUGAAAAAGUGGCACCGAGUCGGUGCUUU
UU-3’

sgRNA2-(U_4Tz_2)
5’-GGGCGAGGAGCUGUUCACCGG**$U_1U_2U_3U_4$-Tz2**AGAGCUAGAAAUAGCAAGUU
AAAAUAAGGCUAGUCCGUUAUCAACUUGAAAAAGUGGCACCGAGUCGGUGCUUU
UU-3’

eGFP-targeting sgRNA, containing 2′-OMe groups (small letters):

sgRNA3
5′-GGGCGAGGAGCUGUUCACCGG**UUUU**AGagcuagaaauagcaaGUU
aAaAuAaggcuaGUccGUUAucAAcuugaaaaagugGcaccgagucggugcuuuuU-3′

sgRNA3-(U_4Tz_1)
5′-GGGCGAGGAGCUGUUCACCGG**UUUU_4-Tz1**AGagcuagaaauagcaaGUU
aAaAuAaggcuaGUccGUUAucAAcuugaaaaagugGcaccgagucggugcuuuuU-3′

sgRNA3-(U_4Tz_2)
5′-GGGCGAGGAGCUGUUCACCGG**UUUU_4-Tz2**AGagcuagaaauagcaaGUU
aAaAuAaggcuaGUccGUUAucAAcuugaaaaagugGcaccgagucggugcuuuuU-3′

<u>VEGFA-targeting sgRNA, containing 2′-OMe groups (small letters):</u>

sgRNA4
5’-GGUGAGUGAGUGUGUGCGUGG**UUUU**AGagcuagaaauagcaaGUU
aAaAuAaggcuaGUccGUUAucAAcuugaaaaagugGcaccgagucggugcuuuuU-3′

sgRNA4-(U_4Tz_1)
5’-GGUGAGUGAGUGUGUGCGUGG**UUUU_4-Tz1**AGagcuagaaauagcaaGUU
aAaAuAaggcuaGUccGUUAucAAcuugaaaaagugGcaccgagucggugcuuuuU-3′

sgRNA4-(U_4Tz_2)
5’-GGUGAGUGAGUGUGUGCGUGG**UUUU_4-Tz2**AGagcuagaaauagcaaGUU
aAaAuAaggcuaGUccGUUAucAAcuugaaaaagugGcaccgagucggugcuuuuU-3′

Capital letters indicate unmodified nucleotides, while small letters correspond to nucleotides containing 2′-OMe groups.

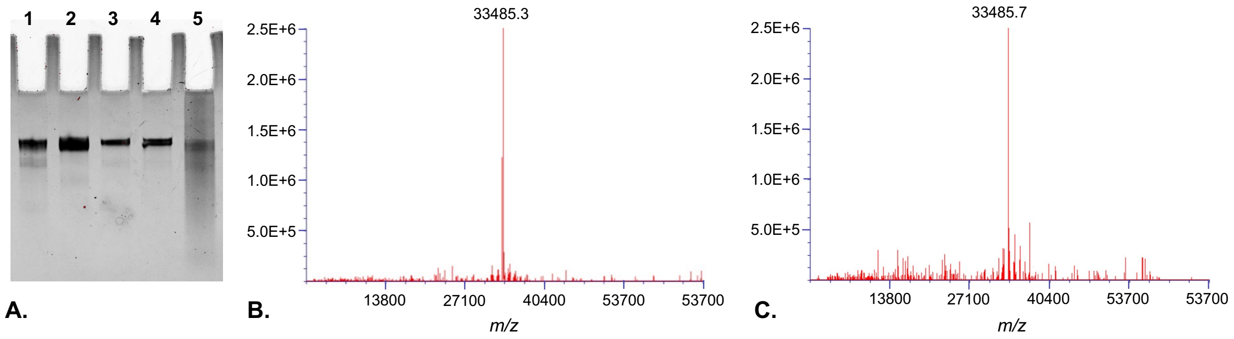

Figure S1. (**A.**) PAGE analysis of purified sgRNAs: *Lane 1*: **sgRNA1-(U$_4$Tz$_2$)**; *Lane 2*: **sgRNA1-(U$_1$Tz$_2$)**; *Lane 3*: **sgRNA1-(U$_4$Tz$_1$)**; *Lane 4*: **sgRNA1-(U$_1$Tz$_1$)**; *Lane 5*: reference. (**B.**) Deconvoluted ESI-MS analysis of **sgRNA1-(U$_1$Tz$_2$)**; (**C.**) Deconvoluted ESI-MS analysis of **sgRNA1-(U$_4$Tz$_2$)**.

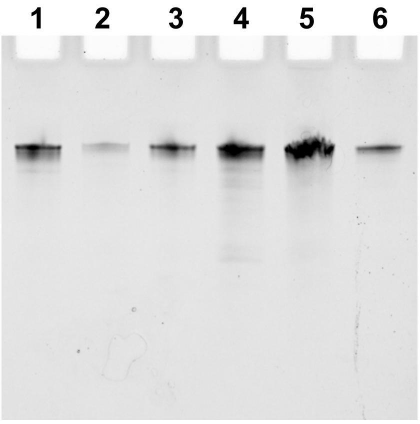

Figure S2. PAGE analysis of purified sgRNAs: *Lane 1*: **sgRNA2-(U$_4$Tz$_2$)**; *Lane 2*: **sgRNA2-(U$_1$Tz$_2$)**; *Lane 3*: **sgRNA2-(U$_4$Tz$_1$)**; *Lane 4*: **sgRNA2-(U$_1$Tz$_1$)**; *Lane 5*: unmodified **sgRNA2**; *Lane 6*: reference.

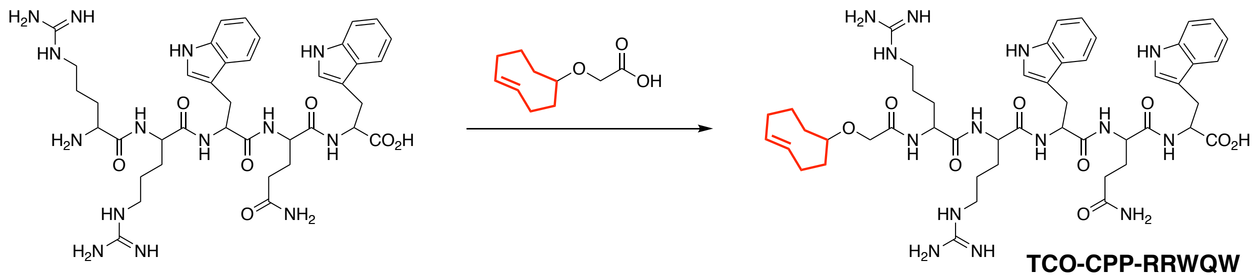

TCO-CPP-RRWQW

Prepared a suspension of HATU (14 mg) in DMF (200 μL). In parallel, prepared a solution of (*E*)-2-(Cyclooct-4-en-1-yloxy)acetic acid (3.3 mg) in NMP (100 μL). Combined the two solutions, added DIPEA (7μL) and 2,6-lutidine (7μL) and stirred for 10 min at rt. Added a solution of **RRWQW** (20 mg, 24 μmol) in DMF (100 μL) and stirred at rt for 2 h. **TCO-CPP-RRWQW** was purified by preparative HPLC and analyzed by analytical HPLC and ESI-MS, shown below. HRMS (ESI) cal'd for $C_{49}H_{69}N_{14}O_9$ $[M+1]^+$ 997.5366; observed 997.5381

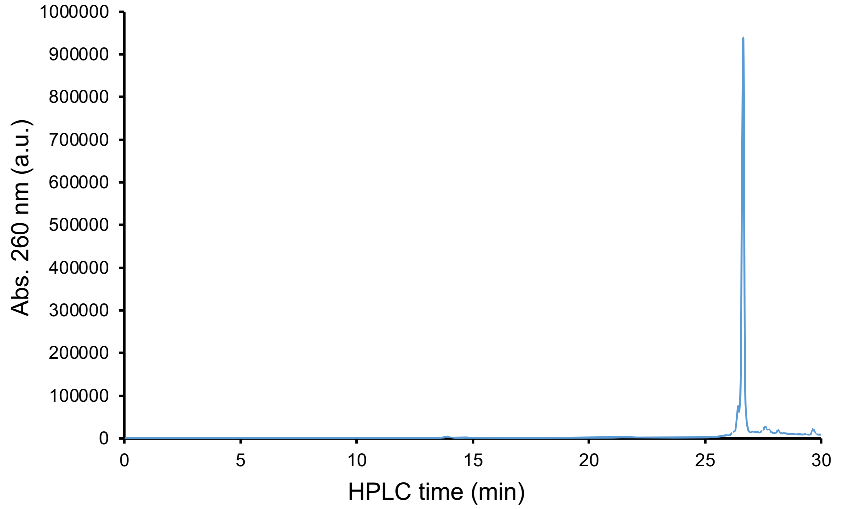

A.

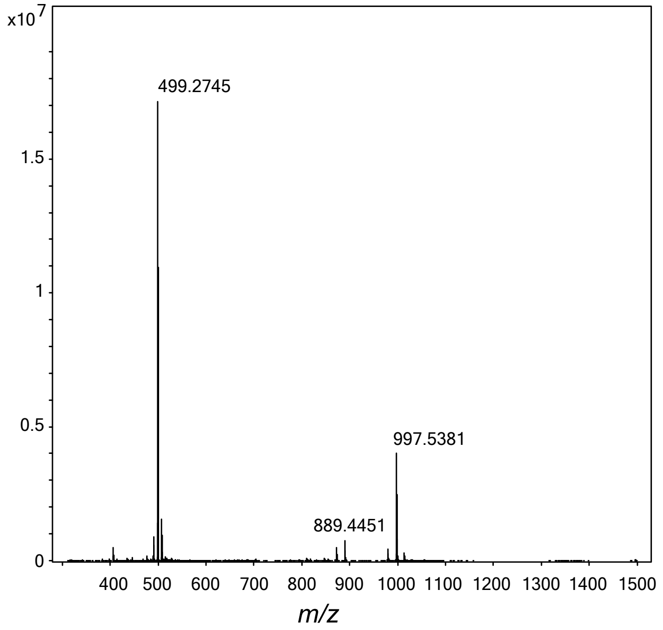

B.

Figure S3. Analytical HPLC (**A.**) and ESI-MS (**B.**) spectra of **TCO-CPP-RRWQW**.

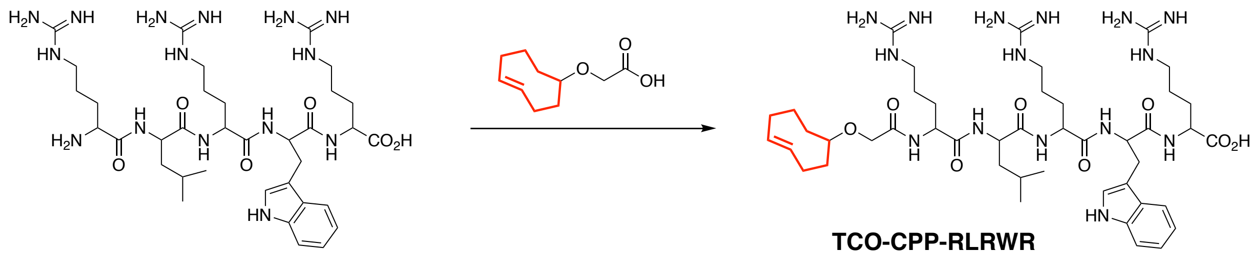

Prepared a suspension of HATU (14 mg) in DMF (200 μL). In parallel, prepared a solution of (*E*)-2-(Cyclooct-4-en-1-yloxy)acetic acid (3.3 mg) in NMP (100 μL). Combined the two solutions, added DIPEA (7μL) and 2,6-lutidine (7μL) and stirred for 10 min at rt. Added a solution of **RLRWR** (20 mg, 25 μmol) in DMF (100 μL) and stirred at rt for 2 h. **TCO-CPP-RLRWR** was purified by preparative HPLC and analyzed by analytical HPLC and ESI-MS, shown below. HRMS (ESI) cal'd for $C_{45}H_{74}N_{15}O_8$ $[M+1]^+$ 952.5839; observed 952.5846

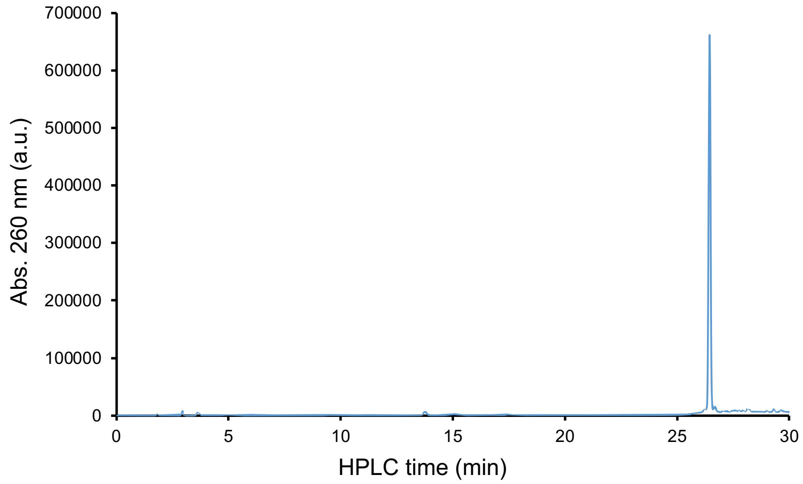

A.

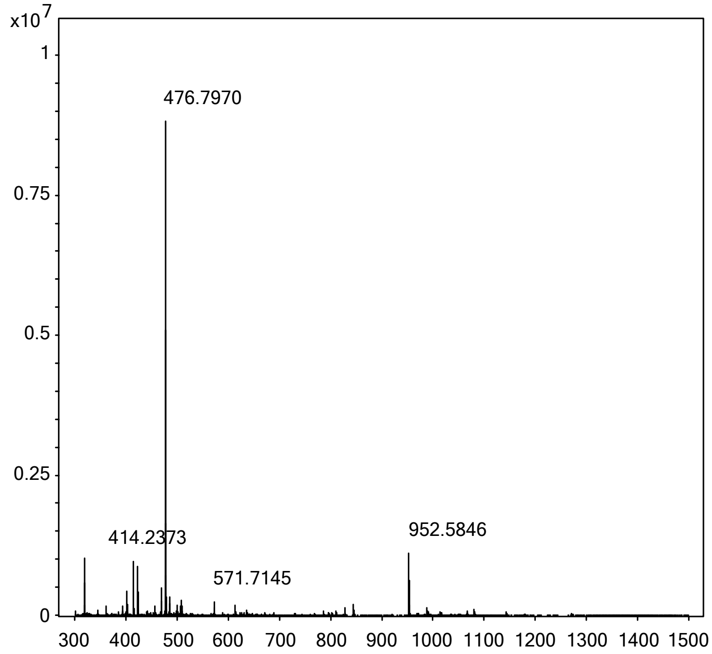

B.

Figure S4. Analytical HPLC (**A.**) and ESI-MS (**B.**) spectra of **TCO-CPP-RLRWR**.

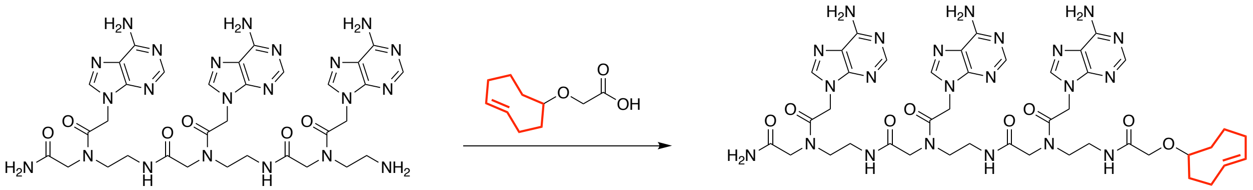

AAA-PNA-TCO

Prepared a suspension of HATU (14 mg) in DMF (200 μL). In parallel, prepared a solution of (*E*)-2-(Cyclooct-4-en-1-yloxy)acetic acid (3.3 mg) in NMP (100 μL). Combined the two solutions, added DIPEA (7μL) and 2,6-lutidine (7μL) and stirred for 10 min at rt. Added a solution of **AAA-PNA** (20 mg, 24 μmol) in DMF (100 μL) and stirred at rt for 2 h. **AAA-PNA-TCO** was purified by preparative HPLC and analyzed by analytical HPLC and ESI-MS, shown below.
HRMS (ESI) cal'd for $C_{43}H_{57}N_{22}O_8$ $[M+1]^+$ 1010.0745; observed 1012.0507

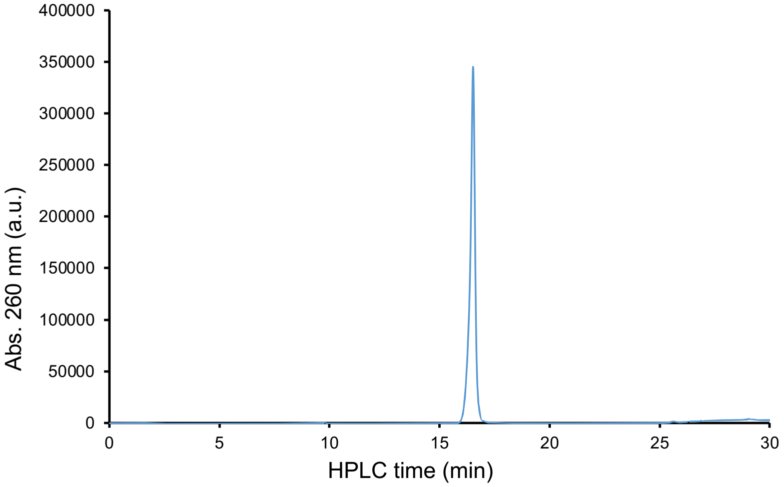

A.

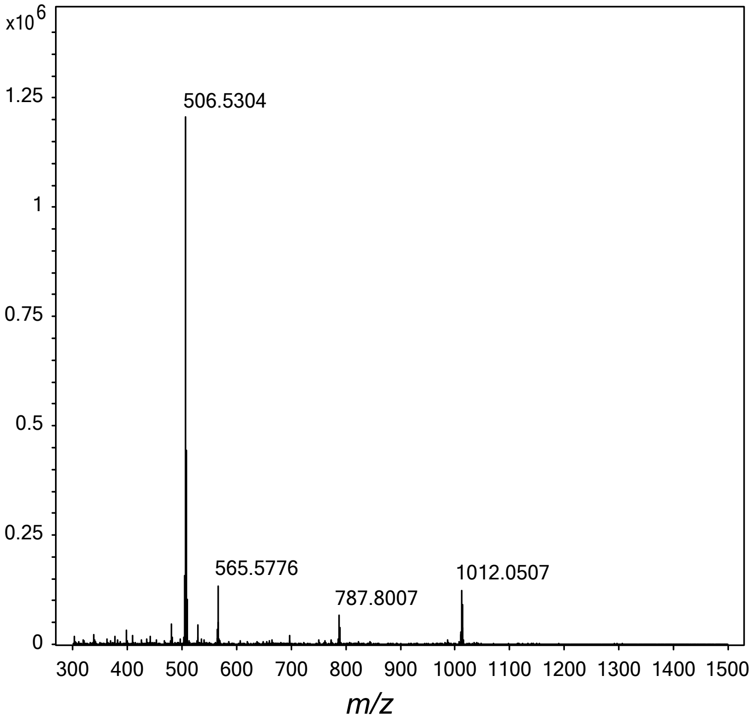

B.

Figure S5. Analytical HPLC (**A.**) and ESI-MS (**B.**) spectra of **AAA-PNA-TCO**.

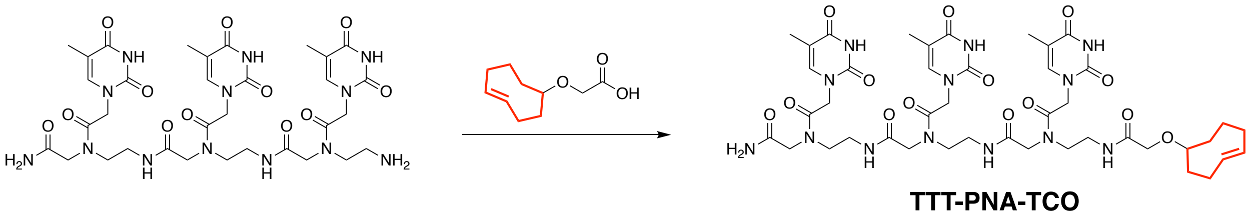

Prepared a suspension of HATU (14 mg) in DMF (200 μL). In parallel, prepared a solution of (*E*)-2-(Cyclooct-4-en-1-yloxy)acetic acid (3.3 mg) in NMP (100 μL). Combined the two solutions, added DIPEA (7μL) and 2,6-lutidine (7μL) and stirred for 10 min at rt. Added a solution of **TTT-PNA** (20 mg, 25 μmol) in DMF (100 μL) and stirred at rt for 2 h. **TTT-PNA-TCO** was purified by preparative HPLC and analyzed by analytical HPLC and ESI-MS, shown below.
HRMS (ESI) cal'd for $C_{43}H_{60}N_{13}O_{14}$ $[M+1]^+$ 982.4377; observed 982.4356

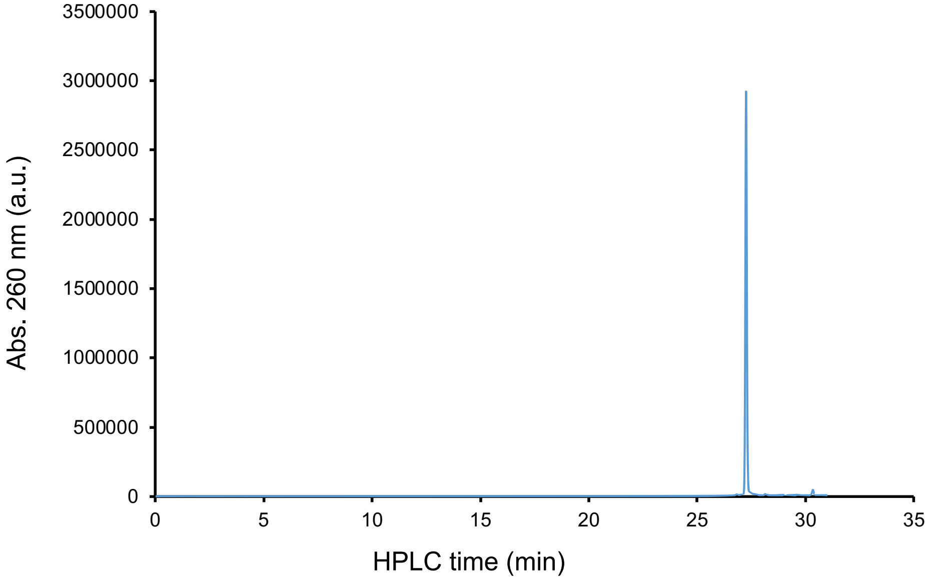

A.

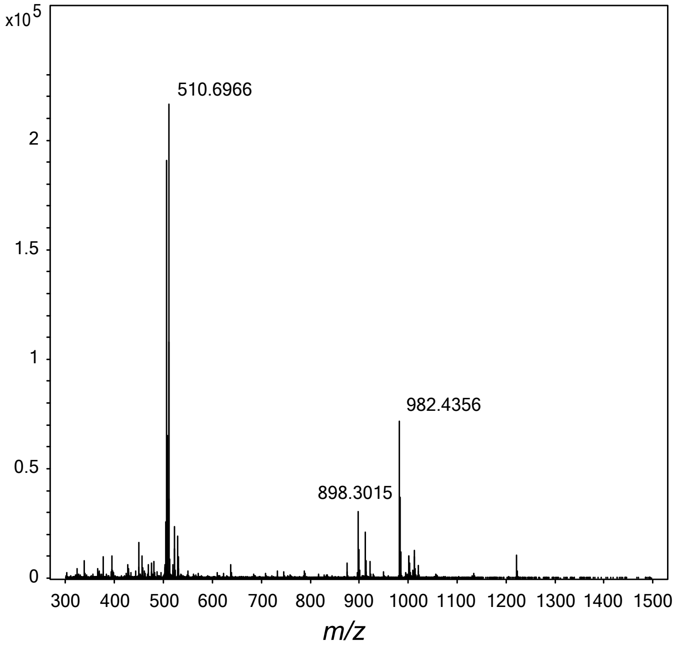

B.

Figure S6. Analytical HPLC (**A.**) and ESI-MS (**B.**) spectra of **TTT-PNA-TCO**.

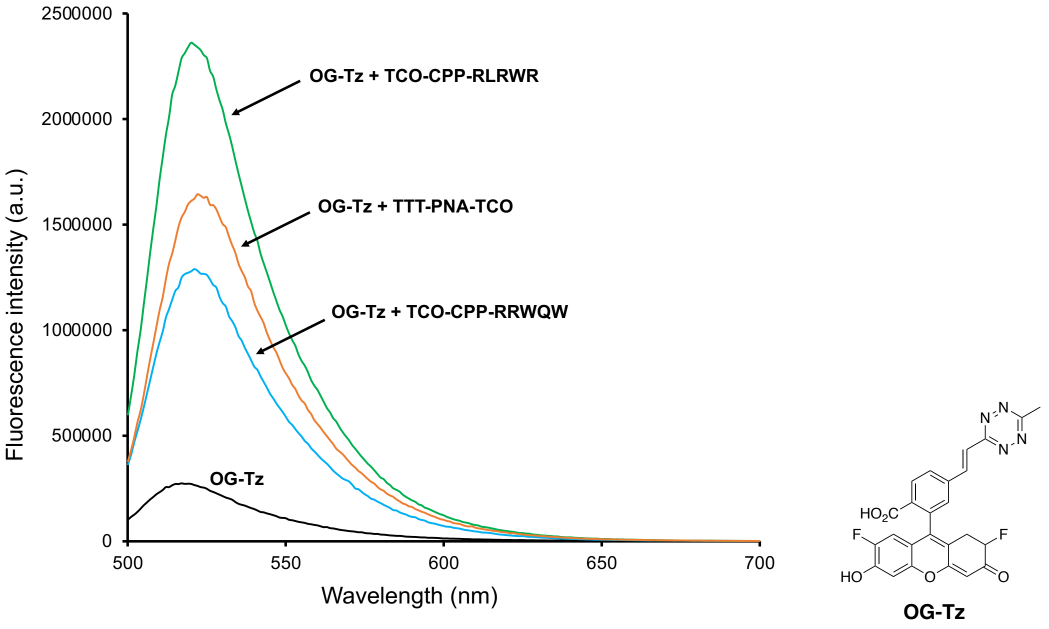

Figure S7. Fluorescence spectra of **OG-Tz** (λ_{ex} = 490 nm) by itself and conjugated to different TCO-modified CRISPR suppressors.

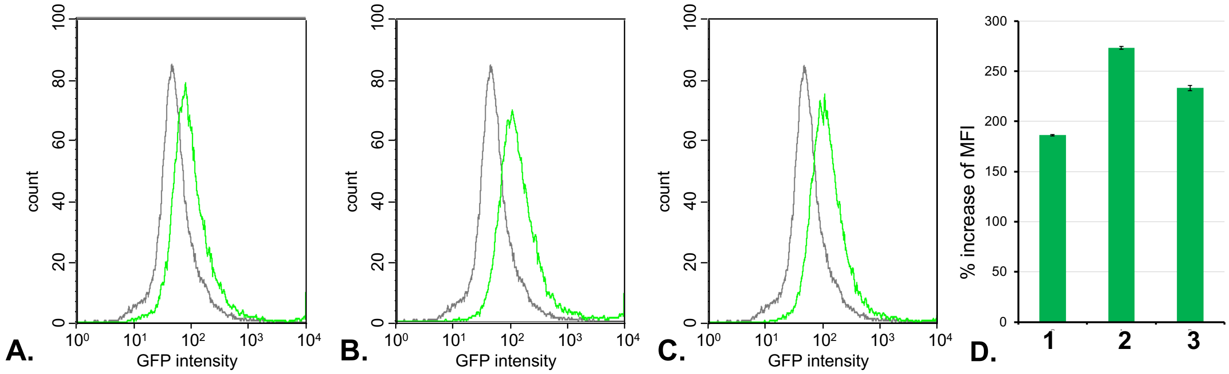

Figure S8. Flow cytometry of HEK293 cells treated with **OG-Tz** alone (black) and with **OG-Tz** and TCO-modified CRISPR suppressors (green). (A) **TTT-PNA-TCO**; (B) **TCO-CPP-RRWQW**; (C) **TCO-CPP-RLRWR**. (D) increase of MFI of **OG-Tz** fluorescence after addition of **TTT-PNA-TCO** (1), **TCO-CPP-RRWQW** (2), **TCO-CPP-RLRWR** (3). All experiments were performed in duplicate. Error bars represent ± s.d.

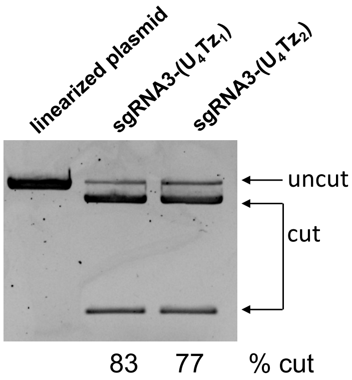

Figure S9. Analysis of CRISPR-Cas9 experiments using agarose gel electrophoresis. (**A**) *Lane 1*: linearized eGFP-N1 plasmid; *Lane 2*: linearized eGFP-N1 plasmid and **sgRNA3-(U_4Tz_1)**; *Lane 3*: linearized eGFP-N1 plasmid and **sgRNA3-(U_4Tz_2)**.

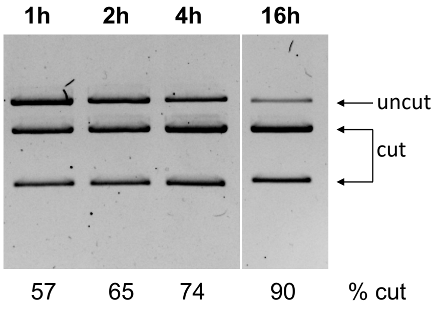

Figure S10. Optimization of Cas9-enabled nuclease activity in the presence of unmodified **sgRNA1** using agarose gel electrophoresis. Linearized pBR322 plasmid was treated with Cas9 and unmodified **sgRNA1** for 1, 2, 4 or 16 h.

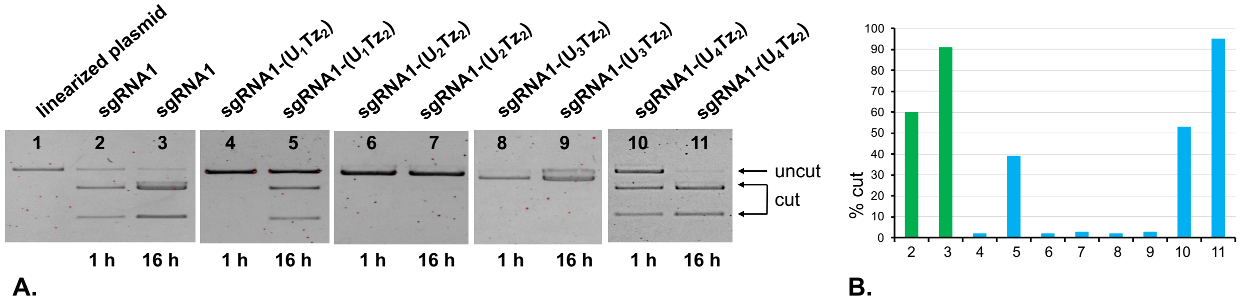

Figure S11. Optimization of Cas9-enabled nuclease activity in the presence of unmodified **sgRNA1**, as well as the experimental ones using agarose gel electrophoresis. (**A**) Linearized pBR322 plasmid was treated with Cas9 and either **sgRNA1** or **sgRNA1-(U_1Tz_2)**, **sgRNA1-**(U_2Tz_2), **sgRNA1-(U_3Tz_2)** or **sgRNA1-(U_4Tz_2)** for 1 or 16 h. (**B**) Bar chart representing the percentage of cut DNA upon treatment with the constructs in part **A**. Lane numbering is the same as in part **A**.

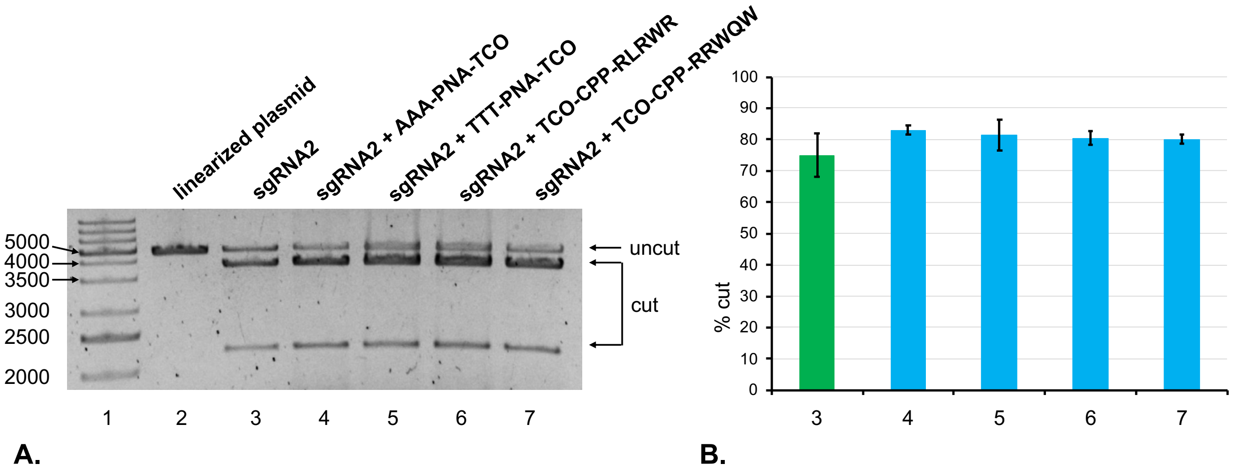

Figure S12. Analysis of impact of different TCO-modified CRISPR suppressors on Cas9-enabled nuclease activity using agarose gel electrophoresis. Linearized eGFP-N1 plasmid was treated with Cas9, **sgRNA 2** and different TCO-modified CRISPR suppressors for 16 h. (**A**) *Lane 1*: ladder; *Lane 2*: linearized eGFP-N1 plasmid; *Lane 3*: linearized eGFP-N1 plasmid treated with Cas9 and **sgRNA 2**; *Lane 4*: linearized eGFP-N1 plasmid treated with Cas9**,** **sgRNA 2** and **AAA-PNA-TCO**; *Lane 5*: linearized eGFP-N1 plasmid treated with Cas9, **sgRNA 2** and **TTT-PNA-TCO**; *Lane 6*: linearized eGFP-N1 plasmid treated with Cas9, **sgRNA 2** and **TCO-CPP-RLRWR**; *Lane 7*: linearized eGFP-N1 plasmid treated with Cas9, **sgRNA 2** and **TCO-CPP-RRWQW**; (**B**) Bar chart representing the percentage of cut DNA upon treatment with the constructs in part **A**. Lane numbering is the same as in part **A**. All CRISPR experiments were performed in duplicate. Error bars represent ± s.d.

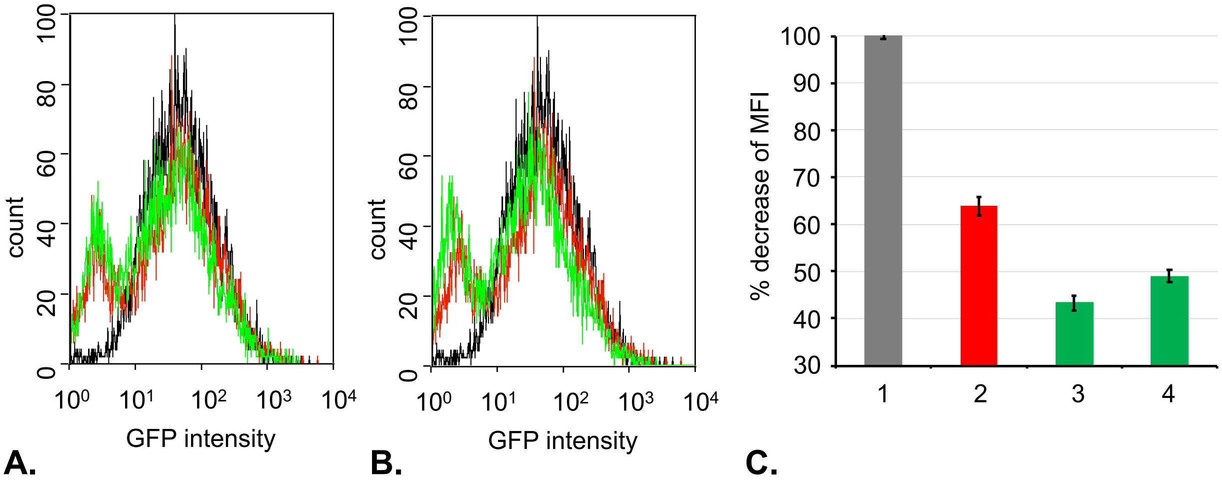

Figure S13. Analysis of GFP expression using flow cytometry. GFP-expressing HEK293 cells were transfected with Cas9 mRNA and either **sgRNA 3** or **sgRNA 3-(U_4Tz_1)** or **sgRNA 3-(U_4Tz_2)** for 72 h. Afterwards, the cells were grown for an additional 48 h. The flow cytometry data is represented as an overlay of histograms (**A.**) the untreated cells (black), cells transfected with Cas9 and **sgRNA 3** (red), cells transfected with Cas9 and **sgRNA 3-(U_4Tz_1)** (green); (**B.**) the untreated cells (black), cells transfected with Cas9 and **sgRNA 3** (red), cells transfected with Cas9 and **sgRNA 3-(U_4Tz_2)** (green). (**C.**) decrease of MFI of GFP relative to the untreated GFP-expressing HEK293 cells. 1) untreated cells, 2) cells transfected with Cas9 and **sgRNA3**, 3) cells transfected with Cas9 and **sgRNA 3-(U_4Tz_1)**, 4) cells transfected with Cas9 and **sgRNA 3-(U_4Tz_2)**. All experiments were performed in duplicate. Error bars represent ± s.d.
